# Enhancing precision harvesting in smart orchards: a light-weight neural network for apple maturity detection

**DOI:** 10.3389/fpls.2026.1820164

**Published:** 2026-05-07

**Authors:** Linna Hu, Penghao Xue, Weixian Zha, Bin Guo, Jia Li, Xingru Chen, Hao Li

**Affiliations:** 1 School of Network and Communication Engineering, Jinling Institute of Technology, Nanjing, China; 2 College of Computer and Information Engineering, Xinjiang Agricultural University, Urumqi, China; 3 Xinjiang Hongqipo Agriculture and Husbandry Investment and Development Group Co., Ltd, Aksu, China

**Keywords:** apple maturity detection, deep learning, HRLN-YOLO, light-weight, smart agriculture

## Abstract

**Introduction:**

Deep learning-based apple maturity detection supports precise management in smart agriculture. However, deployment on resource-constrained edge devices requires minimizing network weights while ensuring accuracy, a challenge compounded by inclement weather and dense fruit clustering in orchard environments.

**Methods:**

To address these challenges, we propose HRLN-YOLO, a lightweight high-efficiency apple maturity detection model that minimizes network weights while ensuring accuracy. Specifically, we design a lightweight backbone HGBackbone to enhance feature extraction and accelerate inference, construct an enhanced neck module RCF_Neck to improve multi-scale feature fusion under occlusion, develop a lightweight detection head LADH-Head to alleviate task conflicts with minimal computational cost, and introduce NWD-Loss to improve localization stability for small-scale targets.

**Results:**

Experiments on the Orchard Apple Maturity Dataset demonstrate that HRLN-YOLO improves mAP@0.5 by 1.7% over the YOLO11n baseline while reducing parameters by 37.3% and computational complexity by 34.9%.

**Discussion:**

The core contribution of this study lies in minimizing network weights while ensuring detection accuracy, providing a practical solution for edge deployment in smart orchard automated harvesting.

## 1 Introduction

Apples, as one of the most widely cultivated high-nutrition fruits worldwide, the precision of their harvesting process directly affects economic benefits and sustainable development potential (Bhavana et al., 2025; Xu and Wang, 2023). The traditional manual harvesting method relies on sensory experience to determine maturity, which has inherent drawbacks such as strong subjectivity, low efficiency, and low standardization. It has become difficult to meet the production needs of large-scale orchards (Musacchi and Serra, 2018; Yuan and Xu, 2025). Moreover, misjudgment of the harvesting time can lead to decreased fruit quality, resource waste, and environmental damage, directly harming economic benefits and restricting agricultural development (Gan et al., 2025). With the application of smart agriculture technologies, promoting the mechanization and automation of orchard harvesting has become the core path to achieve smart agriculture. Automated harvesting systems can precisely grasp the harvesting timing, reduce labor costs, and minimize resource consumption, helping to promote the green and low-carbon development of agricultural production (Chu et al., 2023). However, complex scenarios commonly found in real orchard environments, such as leaf shading, dense fruit overlap, and weather conditions, seriously interfere with the accuracy of maturity detection and target recognition, and remain a major challenge for the application of automated harvesting technology. In addition, under natural growing conditions, maturity differences among fruits are still widespread within orchards. Therefore, developing an efficient apple maturity detection method is of great significance to modern agriculture. On the one hand, it can monitor apple maturity information in real time, collect the maturity information of different apples at different positions in orchards and on fruit trees, provide managers with fruit maturity data in the orchard in a timely manner, and support different distribution strategy decisions for apples of different maturity levels; on the other hand, compared with traditional manual picking strategies, this method can further improve picking efficiency in the orchard and promote the transformation of traditional management methods that rely on manual observation and experience into digitalized and efficient management.

Advances in artificial intelligence have led to the growing application of computer vision in agriculture (Du et al., 2023; Yan et al., 2025). In early studies, machine learning methods were used to detect crops. For example (Yamamoto et al. (2014) proposed a method that combined traditional RGB cam-eras with machine learning to detect tomato fruits. This method, requires manual segmentation of large number of pixels and features, resulting in limited generalization ability and poor environmental adaptability. Later, models based on convolutional neural networks (CNNs) were widely adopted because of the advancement of deep learning (Li N. et al., 2025). An advanced detection model, Faster R-CNN, was proposed in (Bargoti and Underwood, 2017) for fruit detection in orchards, and (Yu et al., 2019) introduced Mask R-CNN, which is improved to detect strawberry based on Faster R-CNN. Although these two-stage algorithms im-prove generalization and robustness compared with traditional methods, they require generating candidate bounding boxes before classification during inference, bringing high computational complexity and slow inference speed. As a classical one-stage algorithm, YOLO (Redmon et al., 2016) performs better because of its excellent precision and quick detection speed, and many researchers have studied its applications in crop detection. For example Liu et al. (2024) proposed an improved YOLOv5-based model to improve inference speed and reduce the number of parameters for apple maturity and diameter detection; (Li Y. et al., 2025) proposed a comprehensive prediction method for apple maturity using structured and unstructured data; (Zhu et al., 2024)presented an improved lightweight model named YOLO-LM based on YOLOv7-tiny, which was applied to detect the maturity of camellia olive fruits in orchards (Wang et al., 2025) proposed an improved NLDETR-YOLO model for fruit and vegetable detection of apples; (Fu et al., 2024) proposed an improved MSOAR-YOLOv10 detection model for detecting occluded apples; (Huang Z. et al., 2025) proposed an improved ORD-YOLO algorithm for citrus maturity detection; (Hao et al., 2025) proposed an improved YOLO-AMAS model to detect the maturity of ‘Jiang’ pomegranate; and (Chen et al., 2025; Qin et al., 2025; Wei et al., 2024) respectively proposed improved YOLO models for detecting cherry tomatoes, tomato maturity, and tomato peduncles. In addition, Sun and Wang (2025) proposed an improved BMDNet-YOLO model for blueberry detection. Moreover, transformer-based RT-DETR (Zhao Y. et al., 2024) models have also been used to crop detection. For example, Wu et al. (2025) proposed an improved RT-DETR model for crop maturity detection by introducing new convolutional modules and attention mechanisms, which im-proved detection precision. In (Yang G. et al., 2025), a novel strawberry maturity detection model, PDSE-DETR, was proposed. The model achieved higher detection precision by optimizing the backbone network structure, attention mechanisms, and loss function. Multi-task learning, as an effective strategy for boosting detection performance, has also been adopted in the field of orchard detection (Zhao J. et al., 2024)employed the YOLOv8 model and took fallen apple detection as an auxiliary task. Through multi-task learning (MTL), the model’s capability to detect apples on trees was further enhanced. This multi-task model demonstrated outstanding robustness and detection precision across various evaluation metrics, yet its performance and practical applicability still have room for further improvement.

The above studies show that deep learning has great significance for agriculture and has broad prospects, especially in crop detection. However, most improvements of these algorithms focus on enhancing detection precision while ignoring model complexity. Considering the deployment of mechanical harvesting, model lightweight de-sign is very important. To address this issue, many studies have focused on exploring lightweight methods. In (Huang Y. et al., 2025), a citrus maturity detection method based on a lightweight RT-DETR was proposed, both the computational complexity and the number of parameters were decreased by 26.5% and 28.1%, respectively. In (Huang Z. et al., 2025), a lightweight pear detection model was proposed, with reductions of 48.47% and 56.2% in the number of parameters and computational complexity, respectively. However, those reduced models still contain 14.28M and 10.24M parameters, and the GFLOPs reach 41.8 and 25.1, indicating that the model structures remain relatively complex. For lightweight improvement, lighter baseline models have greater advantages, and therefore YOLO is more widely adopted. In (Jia et al., 2025), the authors proposed a lightweight YOLO13 model for cherry tomato detection, in which the DPC3K2 module was used to enhance feature extraction capability, and a structured DLAE was introduced to replace standard convolution, thereby further reducing the computational cost. In (Qiu et al., 2025), the model BGWL-YOLO based on YOLO11 was proposed. This model improves multi-scale feature fusion by introducing BiFPN in the neck, re-places standard convolution with GhostConv to reduce redundant computation, introduces new loss function to enhances localization precision. In (Du et al., 2025), YOLO-WAS was proposed. In this model, standard convolution modules are replaced with Adown modules, and a new C2PSA_SCSA module is introduced. In (Yang L. et al., 2025), an improved AAB-YOLO model was proposed for detecting bagged apples. This model enhances feature extraction by introducing the Adown module, strengthens contextual feature fusion using the C3k2_ContextGuided module, and finally adopts an improved detection head and loss function to enhance robustness under occlusion and detection ac-curacy across different scenarios. The above models considerably reduce the number of parameters and computational cost.

Although existing lightweight improvements have achieved clear gains in parameter count and computational cost, there is still room for further improvement in obtaining precise details of apple maturity under the complex conditions of orchard environment. Specifically, limitations still exist in feature detail extraction, multi-scale information fusion, detection robustness, and the localization precision of apple in-stances in complex scenarios such as dense fruits and varying weather and conditions. To solve these issues, this study designs a lightweight high-efficiency apple maturity detection model based on YOLO11. Targeted improvements are made from four aspects: backbone feature extraction, neck feature fusion, detection head design, and regression loss, to better realize a balance between lightweight design and detection precision, thereby enhancing the adaptability of the system to the complex orchard environment during the harvesting process.

The following are summaries of main innovations of this study:

1. To reduce computational redundancy while ensuring effective feature representation, we design a novel lightweight backbone network named HGBackbone. With a more efficient structure, HGBackbone reduces computational cost and memory usage in high-resolution stages while preserving the network’s ability to model detailed features such as texture and edges. In this way, it supports lightweight improvement while still maintaining strong feature expression in apple orchard environments.
2. To improve multi-scale feature utilization under lightweight constraints, the C3k2D module was designed to strengthen feature fusion across different scales. In addition, a new neck structure called RCF_Neck was introduced to alleviate the limitations of the original neck in preserving detailed information and fusing multi-scale features. This design enables the network to better utilize the characteristics of apple targets of different sizes and maturity levels while still maintaining detection accuracy and robustness.
3. To further reduce model complexity for edge deployment while ensuring detection accuracy under complex fruit background conditions, a lightweight LADH-Head is introduced to replace the baseline detection head. This design lowers the computational burden of the model while still improving detection precision.
4. To maintain localization accuracy for small-sized targets under a lightweight detection framework, the NWD-Loss function is adopted to address the sensitivity of IoU-based loss functions (such as CIoU) to position deviation. By using a smoother distance measurement method, it improves the stability of boundary regression for small-scale apple instances.

These improvements enhance the precision of apple maturity detection while making the model more lightweight, which facilitates timely harvesting of ripe fruit to reduce orchard losses and more efficiently promotes smart orchard development.

## 2 Materials and methods

### 2.1 Dataset

**Figure 1:**
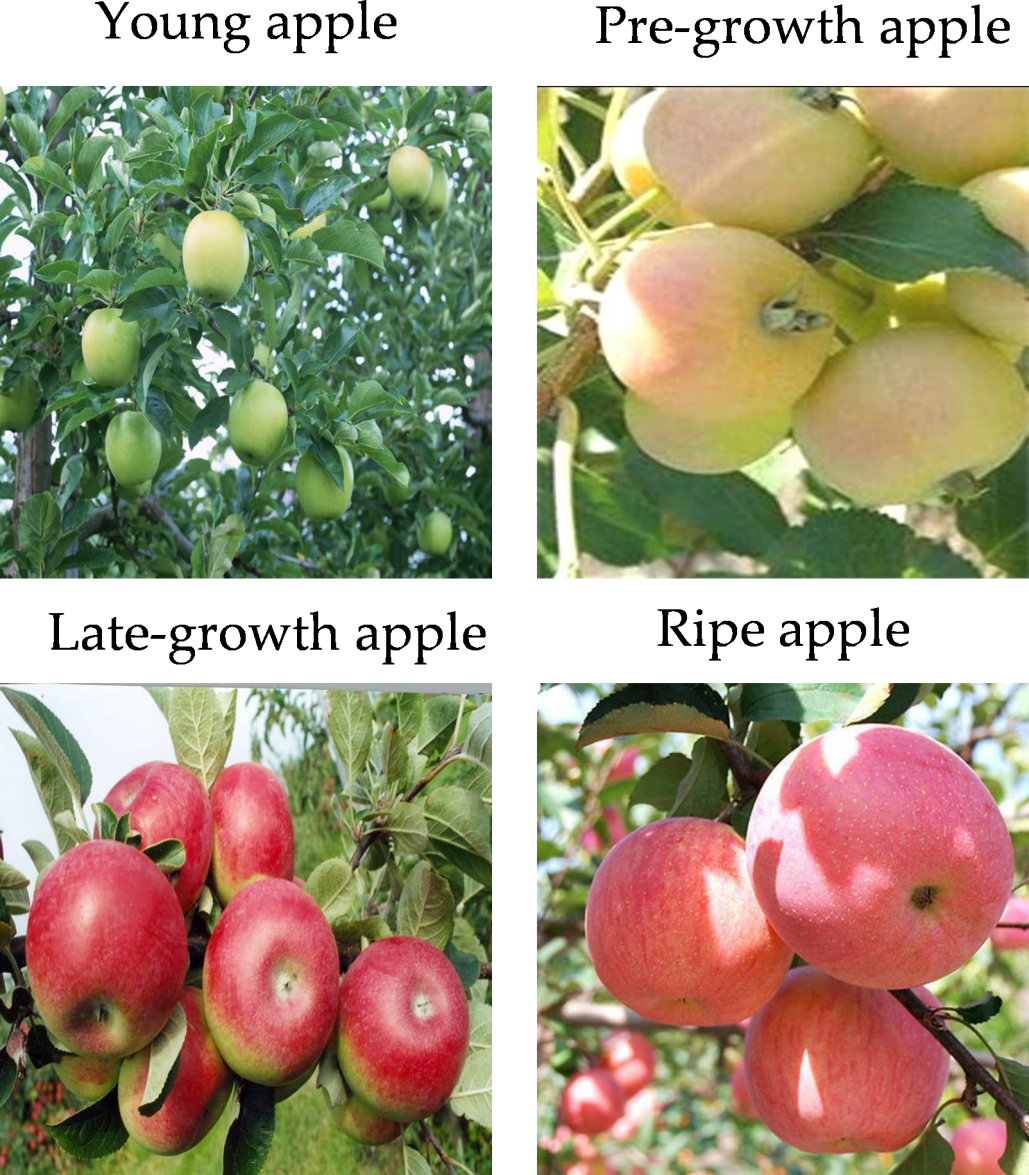
Representative examples of the four different maturity stages in the dataset.

The dataset used in this study is the latest Orchard Apple Maturity Dataset (Tom, 2025) publicly released by TomTom on the FigShare website on 10 July 2025. The dataset contains 2039 images in total. The dataset is split into a training set and a validation set, containing 1618 and 421 images, respectively. All images were captured under real conditions with different lighting and backgrounds. Apples are divided into four categories according to their growth stages: Late-growth apple, Ripe apple, Pre-growth apple, and Young apple. This dataset can directly classify the maturity of apples on trees, which is important for judging whether apples are mature in automatic harvesting scenarios. **Figure 1** displays the sample images from the dataset.

### 2.2 Data augmentation

During model training, this study adopts a unified training strategy that combines framework-built-in augmentation with custom online data augmentation to enhance model generalization performance under complex real-world conditions. First, the model is trained using the following hyperparameter configuration, where the optimizer is initialized with a learning rate of 0.01, and it is linearly decayed to 1% of the initial value. To improve training stability, a 3-epoch warm-up strategy is introduced, during which the learning rate is gradually increased and the momentum parameter is smoothly adjusted from 0.8 to 0.937. A weight decay factor of 5 × 10^−4^ is applied to alleviate overfitting. Regarding the loss function, the weights assigned to bounding box regression, classification, and distribution focal loss (DFL) are 7.5, 0.5, and 1.5, respectively, to balance localization precision and classification performance. In terms of data augmentation, on the one hand, built-in augmentation strategies provided by the Ultralytics framework are adopted. These include HSV color space perturbation with H set to 0.015, S set to 0.7, and V set to 0.4, random translation within ±10%, and scale variation within ±50%, and horizontal flipping (probability 0.5), which simulate different lighting conditions and viewing angles of apples. Mosaic augmentation is enabled (probability 1.0) to enhance model capability for complex backgrounds and multi-scale targets. MixUp and Copy-Paste are not used to avoid semantic confusion between fruits at different maturity stages.

On the other hand, an online data augmentation strategy based on Albumentations is introduced during the training stage and dynamically applied in the preprocessing phase of each training batch. This strategy only includes non-geometric transformations, so no synchronized adjustment of bounding boxes is required. It mainly consists of brightness and contrast perturbation, RGB channel shift, JPEG compression, blur, and noise injection. In this way, the training samples are augmented only during the training stage while maintaining randomness. These augmentation operations are randomly combined according to preset probabilities, which effectively improve sample diversity and model robustness without increasing the original dataset size.

### 2.3 Model selection

**Figure 2:**
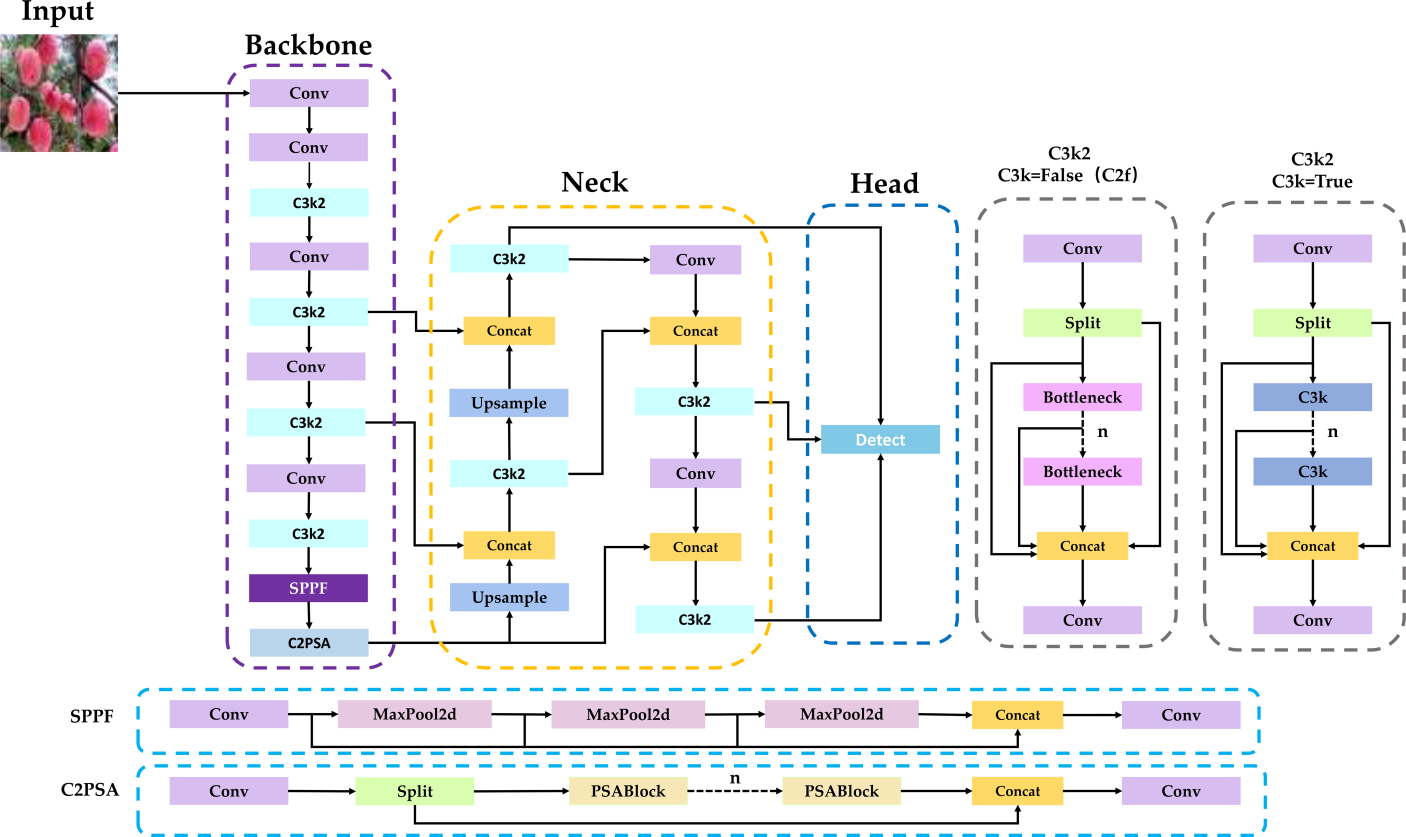
Network structure of YOLO11n.

YOLO11 (Khanam and Hussain, 2024) was proposed by Ultralytics on 30 September 2024. It redefines the limits of object detection with respect to precision, speed, and efficiency. Relative to earlier YOLO releases, YOLO11 introduces notable architectural updates. Specifically, the backbone replaces the C2f module with C3K2 and inserts a C2PSA module after SPPF. In the neck, C3K2 is likewise employed in place of C2f. The overall architecture of YOLO11n is illustrated in **Figure 2**.

In this model, the C3k2 module is a variant of the C2f module. When the C3k module is not used, the original Bottleneck module is used instead, and in this case C3k2 is reduced to C2f. When C3k is enabled, it becomes the complete C3k2 module. In ad-dition, the n shown in the figure is set according to the experimental needs for the C3k and Bottleneck modules.

In tests on the Orchard Apple Maturity Dataset, since YOLO models have five variants with different complexity levels (n, s, m, l, x), we chose the lightest n-level model for the lightweight goal. In **Table 1**, YOLO11n delivers significantly better than YOLOv8n (Jocher et al., 2023), YOLO12n (Tian et al., 2025), and YOLO13n (Lei et al., 2025). After balancing lightweight design and precision, we selected YOLO11n as the baseline model for our improvements.

**Table 1:** Performance comparison among baseline models on the dataset.

Models	mAP@0.5 (%) ↑	mAP@0.5:0.95(%) ↑	FLOPs(G) ↓	Param ↓
YOLOv8n	90.1↑↑	65.4	8.1	3006428
YOLO11n	93.2	68.3	6.3	2582932
YOLO12n	91.4	66.5	5.8	2509124
YOLO13n	92.3	68.4	6.1	2448675

↑ indicates that a higher value is better; ↓ indicates that a lower value is better.

### 2.4 Model improvement: HRLN-YOLO

Although the YOLO11 baseline model has already shown excellent performance on our dataset, in agricultural applications, model parameter quantity and detection performance in specific environments are key factors for real-world deployment on edge devices. In addition, for detection under occlusion or for small targets, the YOLO11 model still has certain disadvantages. Therefore, this study develops a more lightweight apple maturity detector based on YOLO11, termed HRLN-YOLO.

**Figure 3:**
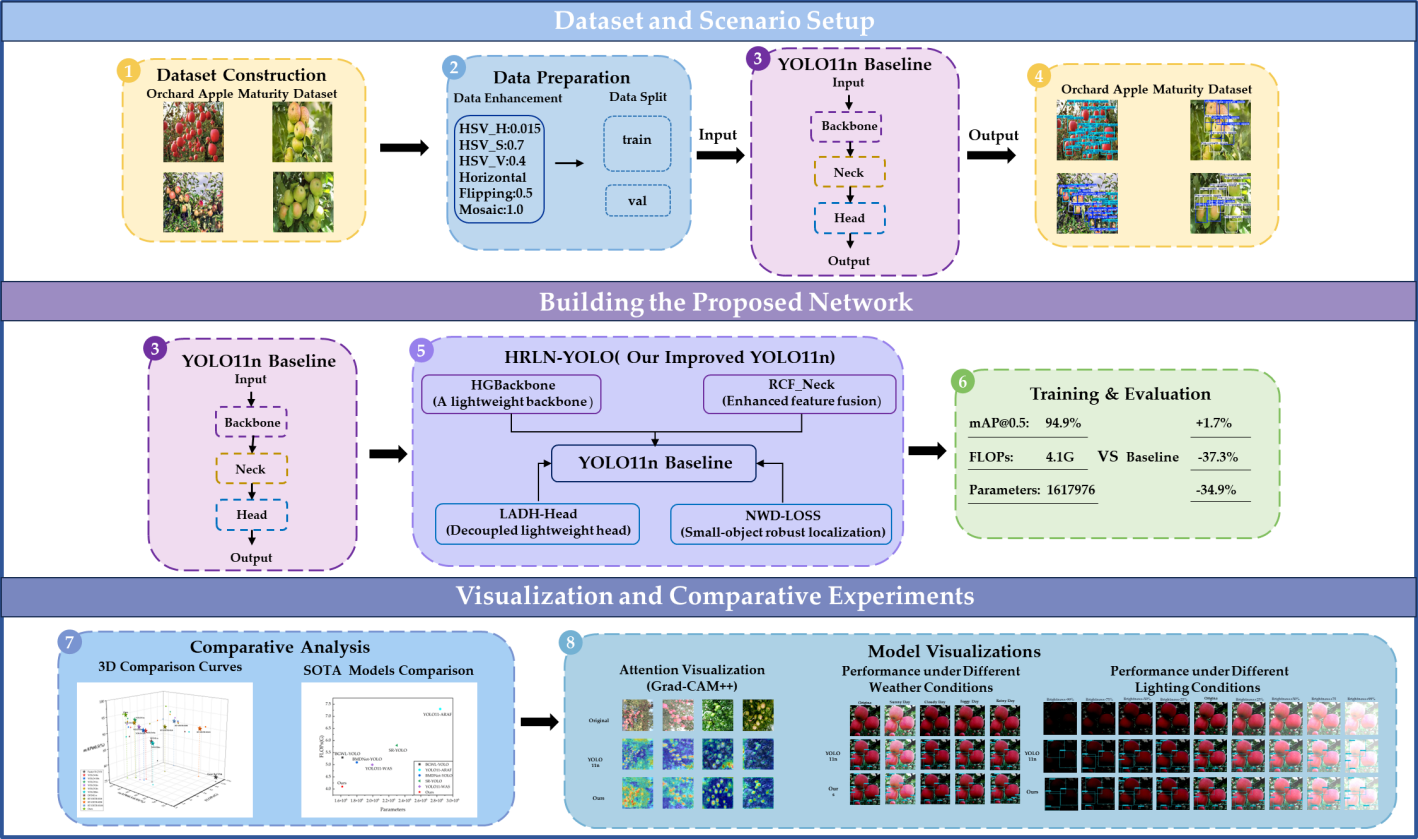
Overall schematic diagram of the proposed HRLN-YOLO framework.

**Figure 4:**
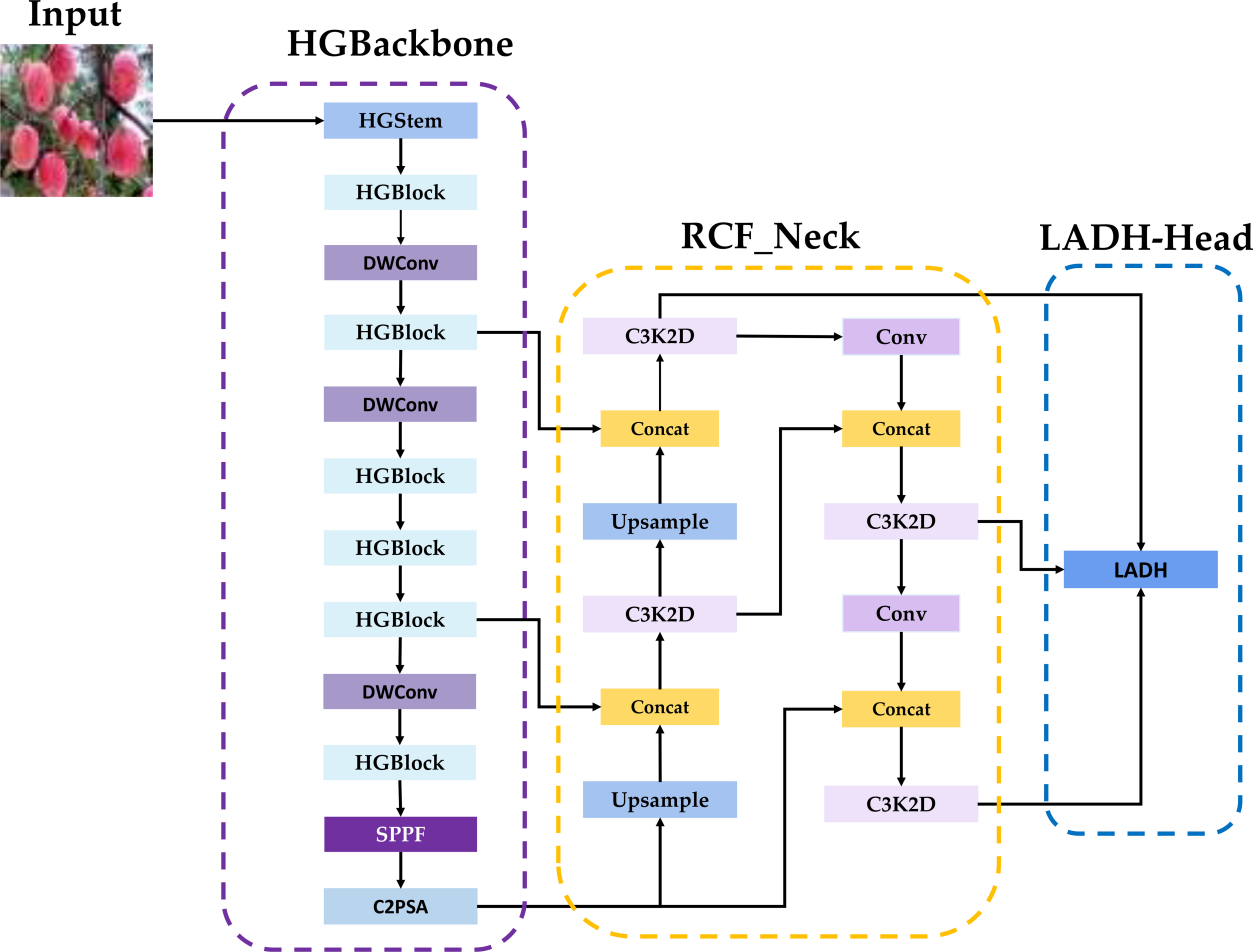
Network structure of HRLN-YOLO.

The overall pipeline is summarized in **Figure 3**. **Figure 3** summarizes the overall pipeline of the proposed method, including dataset, scenario setup, model construction, training evaluation, and visualization-based analyses. The four key improvements (HGBackbone, RCF_Neck, LADH-Head, and NWD-Loss) are demonstrated, and the detailed architecture is provided in **Figure 4**.

We adopted a novel approach to improve the network structure of YOLO11, with the aim of minimizing network weights and model complexity while ensuring detection accuracy. First, we designed a new lightweight backbone network, HGBackbone, whose characteristics of rapidly extracting shallow information and extracting feature information in a stage-wise manner enhance the extraction of maturity-related fine-grained features such as color, texture, and edges, while maximizing the reduction of computational redundancy at the high-resolution stage under the premise of providing high-quality multi-scale features for subsequent stages. Second, we designed a new neck structure, RCF_Neck, which serves as the key component connecting the backbone and the detection head. Its newly designed C3K2D module enhances detail reconstruction and cross-scale semantic interaction, thereby more fully preserving key information such as apple color, texture, and edges, and reducing the complexity of the original neck while ensuring that the detection head receives high-quality fused features. Then, we introduced the lightweight asymmetric decoupled detection head LADH-Head to alleviate the feature coupling conflict between classification and regression tasks and improve the collaborative capability of the model for maturity classification and target localization. While ensuring accuracy, the lightweight convolutions used in this head further contribute to the lightweight design of the model. Finally, we replaced the original loss function with NWD-Loss, enabling the model to still achieve stable bounding box regression under small-target and dense occlusion scenarios. Overall, these designs are not intended merely to pursue structural compression, but rather to effectively optimize the weights and complexity of the original model while ensuring detection accuracy.

#### 2.4.1 HGBackbone

Although the original YOLO11 backbone can accomplish general semantic extraction, it still suffers from a certain degree of computational redundancy at the high-resolution stage, and remains insufficient in extracting fine-grained features such as color, texture, and edges that are required for maturity discrimination. To address this issue, we designed a new lightweight backbone network, HGBackbone, which reduces the computational burden at the high-resolution stage and enhances fine-grained feature extraction capability through stage-wise stacked feature extraction while ensuring feature representation quality as much as possible. The HGBackbone designed in this study is a new lightweight backbone architecture developed based on the HGNetV2 architecture. HGNetV2 is a more lightweight and efficient network proposed by Baidu (Deng et al., 2024; Qian and Zheng, 2025), and this study integrates it into the backbone of YOLO11. This network further strengthens the modeling of fine-grained information, such as local texture and edges, by stacking more 2 × 2 convolutions. It further lowers computational cost by reducing channel width at high-resolution stages, which makes it more suitable for GPU computation (Zhao and Huang, 2024).

**Figure 5:**
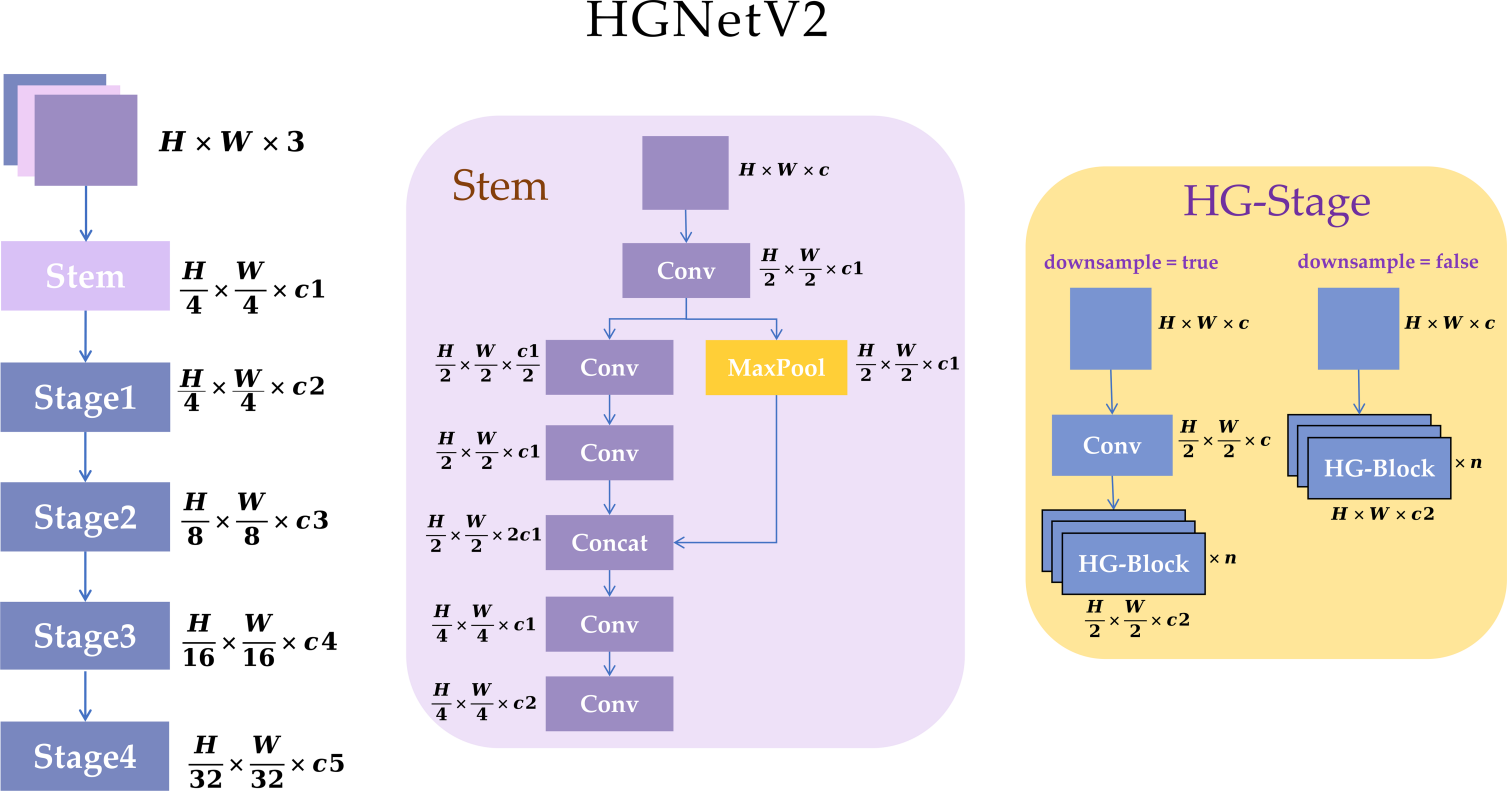
Structure of HGNetV2.

The backbone used in this study mainly consists of HGStem and four resolution stages (Stage1–Stage4). It is then combined with the tail enhancement modules SPPF and C2PSA, forming a new lightweight backbone, HGBackbone. **Figure 5** illustrates the HGNetV2 architecture, where *H* and *W* denote the height and width of the input feature map. *C* represents the number of input channels of the current layer or module, where *n* denotes the number of repetitions of a given structure.

First, HGStem is located at the front of the backbone. It is responsible for quickly extracting basic visual information such as edges and textures at shallow layers. It also completes the initial downsampling through a combination of convolution and pooling, thereby reducing the computational burden caused by high-resolution feature maps in later stages. Then, the network enters the stage-by-stage feature extraction process. Stage1 stacks several HGBlocks while keeping the resolution unchanged, to further refine shallow fine-grained textures and local structures. When moving to the next scale, DWConv is used as a downsampling transition layer between stages, which gradually reduces the spatial resolution of the feature map as well as enters Stage2, Stage3, and Stage4, forming hierarchical semantic representations from shallow to deep layers.

**Figure 6:**
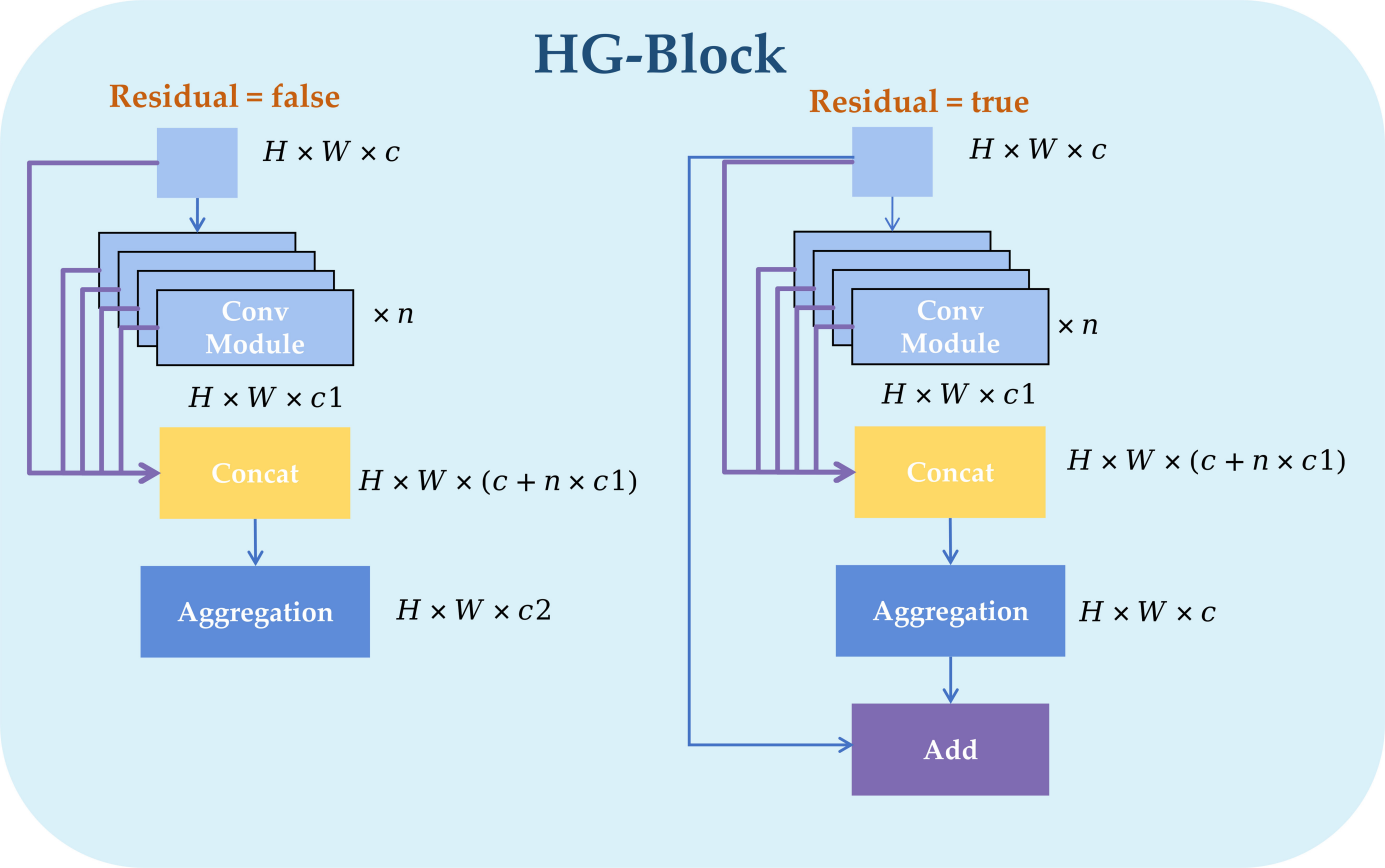
Structure of HGBlock.

**Figure 7:**
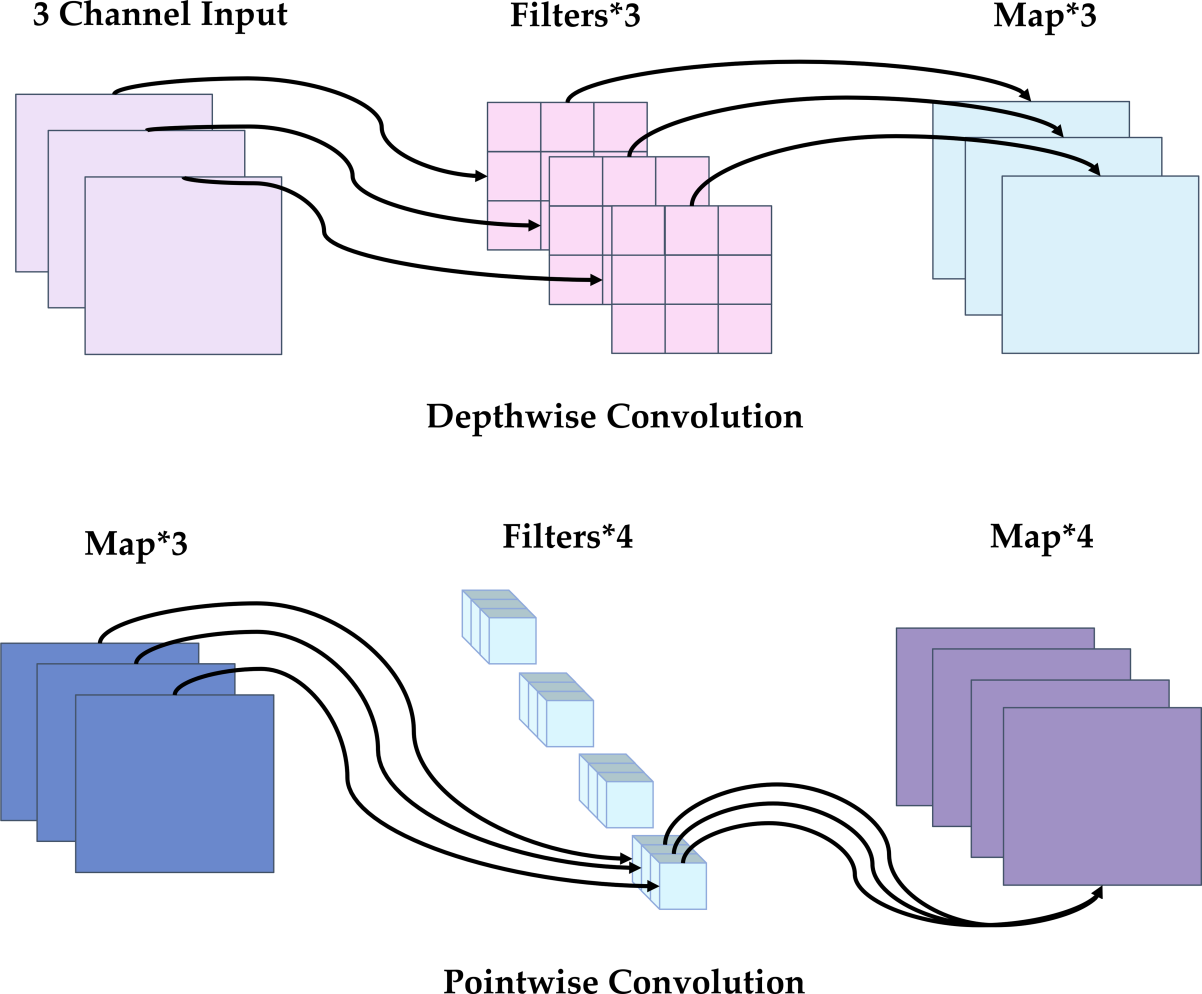
Structure of DWConv.

Each stage uses HGBlock as the core unit. HGBlock generates and fuses multiple groups of intermediate features step by step. **Figure 6** illustrates the HGBlock architecture. These features are concatenated (Concat) along the channel axis, and then an output representation is obtained through aggregation and compression (Aggregation). When residual connection is enabled (Residual = true), the module output is Add to the input to improve gradient propagation and training stability. On the other hand, the lightweight mechanism of DWConv (Srivastava and Sarawadekar, 2020) is shown in the **Figure 7** (DWConv illustration). Its working process can be understood as follows: in the depthwise convolution stage, a *k* × *k* convolution kernel is applied to each input channel separately (the kernel count matches the input-channel number, while the output-channel number stays unchanged). Then, a 1 × 1 pointwise convolution performs a linear combination along the channel dimension to achieve cross-channel fusion and map features to the required number of output channels. As a result, DWConv can significantly reduce parameter count and FLOPs while keeping spatial representation ability as much as possible.

Finally, SPPF is appended to the backbone tail to enrich multi-scale contextual cues, and C2PSA is used to further strengthen the responses of key regions/channels. This provides more discriminative high-level semantic representations for the subsequent feature fusion in the Neck and for maturity classification and detection in the Head.

In conclusion, HGBackbone uses HGStem to complete the rapid extraction of shallow fine-grained information, and combines the progressive feature accumulation and dense concatenation mechanism of multi-stage HGBlocks, thereby effectively reducing the computational redundancy of the traditional backbone at the high-resolution stage while maintaining strong feature representation capability. At the same time, DWConv is used to achieve lightweight downsampling transitions between stages, enabling the network to gradually build hierarchical representations from shallow textures to high-level semantics under relatively low computational cost. Therefore, HGBackbone not only has better lightweight characteristics, but can also more fully preserve the key information required for maturity discrimination, such as color, texture, and edges. For the apple maturity detection task, this module can provide a higher-quality multi-scale feature foundation for subsequent neck fusion and detection prediction, thereby enhancing the model’s ability to perceive subtle visual differences among apples at different maturity stages and improving its adaptability to complex orchard environments.

#### 2.4.2 RCF_Neck

At the feature fusion stage, the original three-scale FPN+PAN topology in YOLO tends to weaken low-level discriminative details during multi-scale fusion under scenarios involving occlusion, dense overlap, small targets, and complex backgrounds, thereby affecting maturity classification performance. To address this problem, we designed a new neck structure, RCF_Neck, as well as a new module, C3K2D.This enables stronger detail reconstruction and multi-scale semantic interaction while maintaining lightweight constraints, greatly enhancing the fusion capability for apple color or texture details and providing high-quality fused features for the detection head. C3k2D uses Bottleneck_Converse as the basic unit and introduces Converse2D (Huang X. et al., 2025), which is based on inverse-problem modeling, within the feature-fusion stage. This allows the neck network to have stronger detail reconstruction and information fidelity under lightweight constraints, thereby improving maturity discrimination in small-target and occlusion scenes.

The principle of Converse2D can be understood from the mathematical modeling of the inverse convolution problem. Let the high-resolution latent feature be *X*. After applying a depthwise separable convolution kernel *K* and downsampling with stride s, the observed low-resolution feature *Y* is obtained. This forward degradation process can be expressed as Equation 1:

(1) Y=(X∗K)↓s

Our goal is to recover the latent high-resolution feature *X* from the observed output *Y*, along with the convolution kernel *K* and stride *s*. Formally, this can be written as Equation 2:

(2) X=T(Y,K,s)

Here, T(·) represents the inverse mapping of convolution and downsampling, that is, the Converse2D operation used in this study. Since downsampling introduces irreversibility, this problem is usually ill-posed, and a direct inversion is unstable. Therefore, we formulate it as a regularized least-squares problem. First, we consider the data-consistency term:

(3) $$\underset{X}{min}{\left\|(X*K)↓s-Y\right\|}_{F}^{2}$$

That is, by minimizing the error between the convolution-and-downsampling result generated from *X* and the observed *Y*, we approximate the original input. However, directly minimizing Equation 3 is often unstable. Therefore, we introduce a quadratic regularization term to constrain the solution space:

(4) $$\underset{X}{min}{\left\|(X*K)↓s-Y\right\|}_{F}^{2}+\lambda {\left\|X-X_{0}\right\|}_{F}^{2}$$

Here, $∥\cdot {∥}_{F}$ denotes the Frobenius norm, λ > 0 is the regularization coefficient, and *X*_0_ is a prior initial estimate of (*X*) (it can be obtained by upsampling (*Y*), for example, by nearest-neighbor interpolation). The first term ensures that the reconstructed result can explain the observed (*Y*) after the forward degradation process, and the second term suppresses noise amplification and numerical divergence in irreversible frequency bands, thereby improving the stability of the solution.

**Figure 8:**
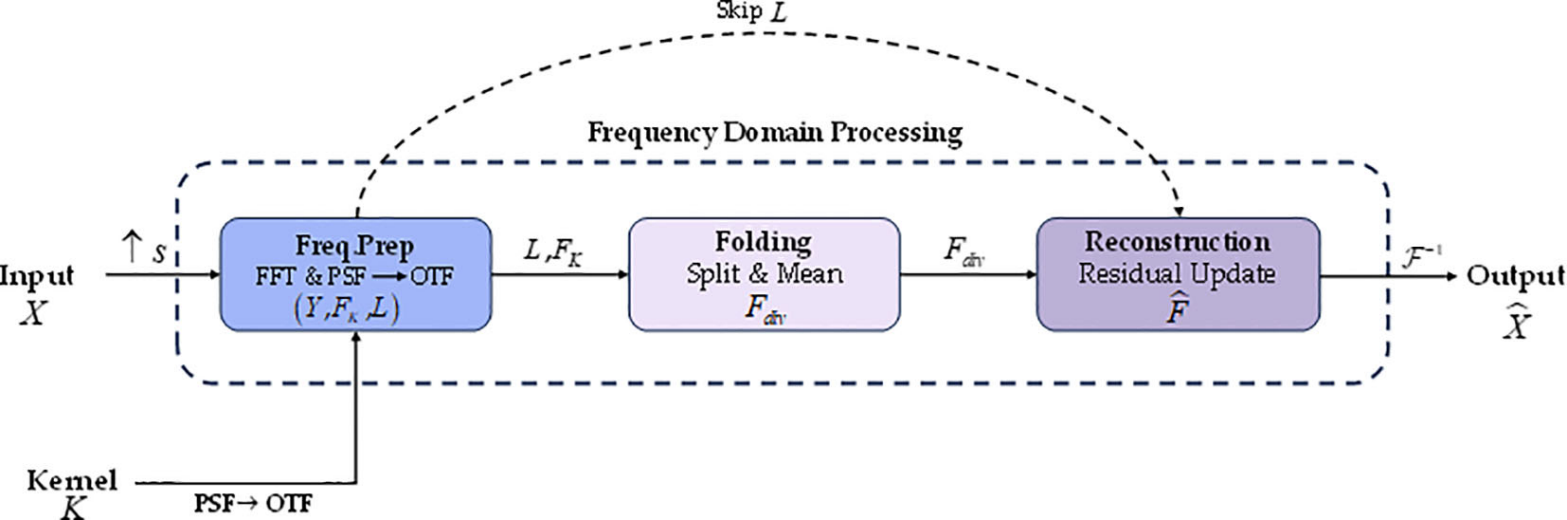
Structure of Converse2D.

Assuming circular boundary conditions, convolution can be diagonalized in the frequency domain, and thus Equation 4 can yield a closed-form solution in the frequency domain. **Figure 8** illustrates the frequency-domain computation framework of Converse2D, where the input is the observed feature *X*, and ↑*_s_* denotes upsampling by a factor *s* to the target resolution. The convolution kernel K is converted from PSF to OTF and represented in the frequency domain as $F_{K}=\text{ℱ}\left(K\right)$, where ℱ(·) and *F*^−1^(·) denote FFT and IFFT, respectively. In the Freq.Prep stage, the right-hand term *L* in the frequency domain (jointly formed by the data-consistency term and the prior term) is constructed and, together with *F_K_*, is passed to the subsequent solver; the dashed “Skip *L*” indicates that this term is directly fed as a residual shortcut for update. Folding (Split&Mean) partitions the spectrum into *s*^2^ phase blocks according to an *s* × *s* grid and aggregates them to explicitly handle the aliasing introduced by stride-based downsampling, producing the low-resolution frequency-domain quantity *F_div_*. In the Reconstruction stage, a residual-form frequency-domain update is completed based on *F_div_*, *F_K_*, and *L* to obtain $\hat{F}$; finally, the reconstructed feature $\hat{X}$ is obtained via ${\text{ℱ}}^{-1}$. This pipeline achieves stable reconstruction through “closed-form frequency-domain updates + explicit aliasing modeling”, enabling the fusion node to preserve more high-frequency discriminative cues such as texture and edges under lightweight constraints.

Structurally, C3k2D inherits the CSP cross-stage residual fusion framework of C3k2. It replaces the stacked Bottleneck blocks with Bottleneck_Converse, so that detail reconstruction capability based on Converse2D is introduced without significantly increasing parameter count and computational overhead. In the apple maturity detection task, feature information such as the color and texture of the apple surface is extremely important. Compared with the simple concatenation and interpolation-based upsampling in the original YOLO11, RCF_Neck, relying on the C3K2D module and the Converse2D inverse-problem reconstruction mechanism, upgrades feature fusion into a reconstruction-enhanced fusion process, fundamentally restoring the high-frequency discriminative information lost during downsampling, thereby improving the feature integrity of occluded fruits and small targets and transmitting more stable discriminative information to the detection head.

#### 2.4.3 LADH detection head

**Figure 9:**
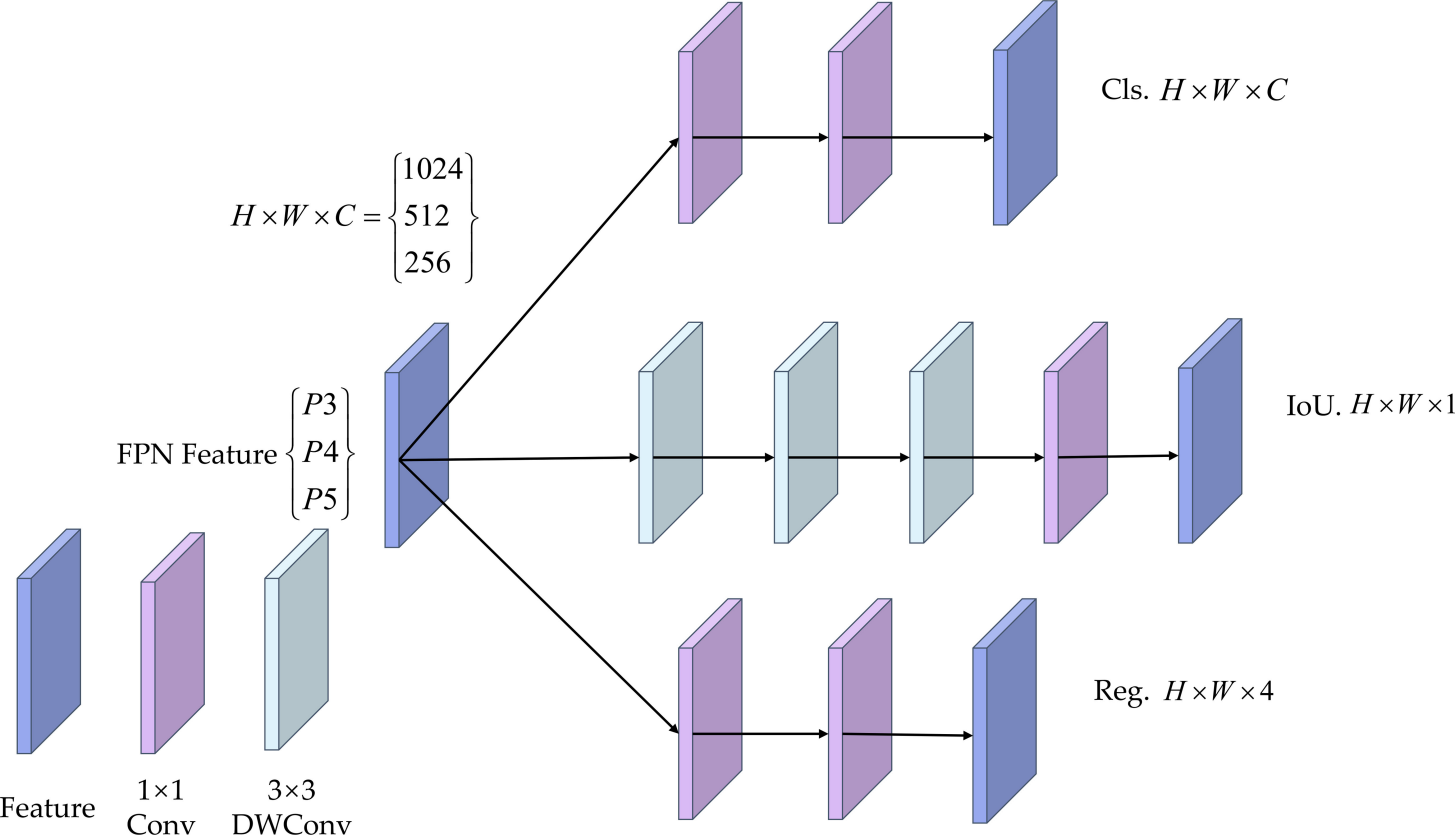
Structure of LADH-Head.

To address the problem that the original YOLO11 detection head shares too much feature representation between classification and regression tasks, which may lead to task conflict, we introduced the lightweight asymmetric decoupled detection head LADH-Head (Zhang et al., 2023), based on the original detection head. LADH-Head divides the FPN features at each scale (P3, P4, and P5) into three parallel branches according to tasks: Cls, IoU, and Reg. In the IoU branch, a deeper convolutional stack is adopted to enlarge the receptive field and improve the modeling of localization quality. To further achieve lightweight design, this branch replaces the standard 3 × 3 convolution with 3 × 3 depthwise separable convolution (DWConv). In this way, it lowers parameter count and computational cost while preserving a certain level of representational capacity, and it improves inference efficiency. The final outputs are the classification prediction *H* × *W* × *C*, the bounding box regression *H* × *W* × 4, and the IoU prediction *H* × *W* × 1. In apple maturity detection, this lightweight decoupled design helps improve detection stability under complex backgrounds and occlusion while controlling computational cost. **Figure 9** shows the structure of LADH-Head. Here, *H* × *W* is the feature map resolution at this scale, *C* is the number of classes, Cls, Reg, and IoU are the outputs of the three branches, p3, p4, and p5 correspond to the three-scale inputs, and 1024, 512, and 256 are the input channel numbers at each scale.

Apple maturity detection has obvious dual attributes: on the one hand, it is necessary to determine the maturity stage according to semantic features such as color and texture; on the other hand, it is also necessary to accurately regress the target position to ensure that classification is performed on the correct fruit region. If the classification and regression branches are too strongly coupled, one task may suppress the optimization of the other. The lightweight asymmetric decoupled detection head LADH-Head divides classification, IoU estimation, and bounding box regression into different branches, and reduces additional overhead through DWConv. Compared with the original YOLO11 detection head, LADH-Head enables the classification branch to focus more on maturity-related color and texture information, while allowing the regression and IoU branches to focus more on edge geometry and localization quality, thereby making it more suitable for simultaneously accomplishing maturity classification and target localization in complex orchard backgrounds.

#### 2.4.4 NWD-loss function

The CIoU in YOLO11 is an IoU-based metric. When the fruit targets are small and there are dense occlusions and overlaps, the targets are often composed of only a few pixels. In this case, even a slight pixel-level shift of the predicted box can cause a sharp drop in IoU, which makes the regression supervision unstable. To provide a smoother regression signal for small-scale targets, this study adopts the Normalized Wasserstein Distance (NWD) (Wang et al., 2021) as a similarity metric and embeds it into the bounding box regression loss.

Specifically, a horizontal bounding box *R* = (*c_x_*, *c_y_*, *w*, *h*) is modeled as a two-dimensional Gaussian distribution *N*(*μ*, Σ), shown as Equation 5:

(5) $$\mu =\left[\begin{array}{c} c_{x}\\ c_{y} \end{array}\right],\Sigma =\left[\begin{array}{cc} \frac{w^{2}}{4} & 0\\ 0 & \frac{h^{2}}{4} \end{array}\right]$$

Assume the predicted and ground-truth boxes are represented by Gaussian distributions *N_a_* and *N_b_*, respectively. The second-order Wasserstein distance between them can be simplified in the horizontal-box case as Equation 6:

(6) $$W_{2}^{2}(N_{a},N_{b})={\left\|\left[\begin{array}{l} c_{x_{a}}\\ c_{y_{a}}\\ \frac{w_{a}}{2}\\ \frac{h_{a}}{2} \end{array}\right]-\left[\begin{array}{l} c_{x_{b}}\\ c_{y_{b}}\\ \frac{w_{b}}{2}\\ \frac{h_{b}}{2} \end{array}\right]\right\|}_{2}^{2}$$

Since $W_{2}^{2}$ is a distance rather than a similarity measure, NWD uses an exponential form to normalize it into a [0, 1] similarity:

(7) $$NWD(N_{a},N_{b})=\exp\left(-\frac{\sqrt{W_{2}^{2}(N_{a},N_{b})}}{C}\right)$$

In Equation 7, *C* is a dataset-dependent constant, typically set to the mean absolute size of the target bounding boxes in the dataset. Finally, the regression loss is defined as Equation 8:

(8) $$L_{NWD}=1-NWD\left(N_{a},N_{b}\right)$$

When the CIoU loss function in YOLO11 is replaced with *L_NWD_* (NWD-Loss), the bounding box IoU is transformed into a continuous probabilistic distribution distance measure. Gaussian modeling eliminates hard boundaries, Wasserstein distance provides linearly smoothed variation, and exponential normalization keeps the function differentiable everywhere. As a result, bounding box regression under small-target and occlusion scenarios can obtain stable and continuous gradient signals, thereby greatly improving the detection performance of small-sized apples or densely distributed occluded apples in images.

### 2.5 Evaluation metrics

To systematically evaluate the detection performance and computational efficiency of the improved YOLO11 model in apple maturity detection, this study selects evaluation metrics from two aspects: detection precision and model complexity. The evaluation metrics mAP@0.5, mAP@0.5:0.95, Precision, Recall, F1-score, Parameter count, Gloating-point operations (GFLOPs), and Inference Speed (FPS). The definitions are provided below.

(9) $$mAP@0.5=\frac{1}{N}\sum_{i=1}^{N}\int_{0}^{1}P_{i}\left(R\right)dR$$

In Equation 9, *P_i_* (*R*) represents the precision of the *i* class at recall *R*. In the multi-class case, mAP@0.5 is computed as the mean *AP* over all categories and then, the expression of mAP@0.5: 0.95 is as shown in Equation 10:

(10) $$mAP@0.5:0.95=\frac{1}{10}\sum_{t=0.5}^{0.95}mAP@t$$

(11) $$Precision=\frac{TP}{TP+FP}$$

(12) $$\text{Recall}=\frac{TP}{TP+FN}$$

(13) $$F1=\frac{2\times \text{Precision}\times \text{Recall}}{\text{Precision}+\text{Recall}}$$

In Equations 11–13, where TP, FP, TN, and FN represent the numbers of true positives, false positives, true negatives, and false negatives, respectively, and n denotes the total number of classes.

(14) $$Params=\sum_{l=1}^{L}\left(C_{l}^{in}\times C_{l}^{out}\times K_{l}^{2}\right)$$

In Equation 14, *L* represents the number of network layers. $C_{l}^{in}$ and $C_{l}^{out}$ denote the input and output channel numbers of the *l* layer, respectively, and *K_l_* represents the convolution kernel size. In practical experiments, the number of parameters is usually reported in millions (M).

(15) $$FLOPs_{l}=2\times H_{l}\times W_{l}\times C_{l}^{in}\times C_{l}^{out}\times K_{l}^{2}$$

In Equation 15, *H_l_* and *W_l_* denote the feature-map height and width, respectively, the factor of 2 corresponds to one multiply–accumulate (MAC) operation. The expression of GFLOPs is shown in Equation 16.

(16) $$GFLOPs=\frac{1}{10^{9}}\sum_{l=1}^{L}FLOPs_{l}$$

(17) $$FPS=\frac{N}{T}$$

In Equation 17, *N* denotes the number of images used for inference, and *T* denotes the total inference time in seconds. A higher *FPS* reflects stronger real-time detection capability.

## 3 Results and analysis

### 3.1 Experimental setup

The hardware and software settings used in this study are summarized in **Table 2**.

**Table 2:** Experimental configuration and environment parameters.

Environmental parameter	Parameter value
Graphics Card	NVIDIA GeForce RTX 4090D Laptop GPU
Memory	8GB
RAM	24G
Pytorch	2.2.2+cuda12.1
Python	3.10.16
Batch size	32
Optimizer	SGD
Img Size	640*640
Epochs	600
patience	30

### 3.2 Ablation experiments

**Figure 10:**
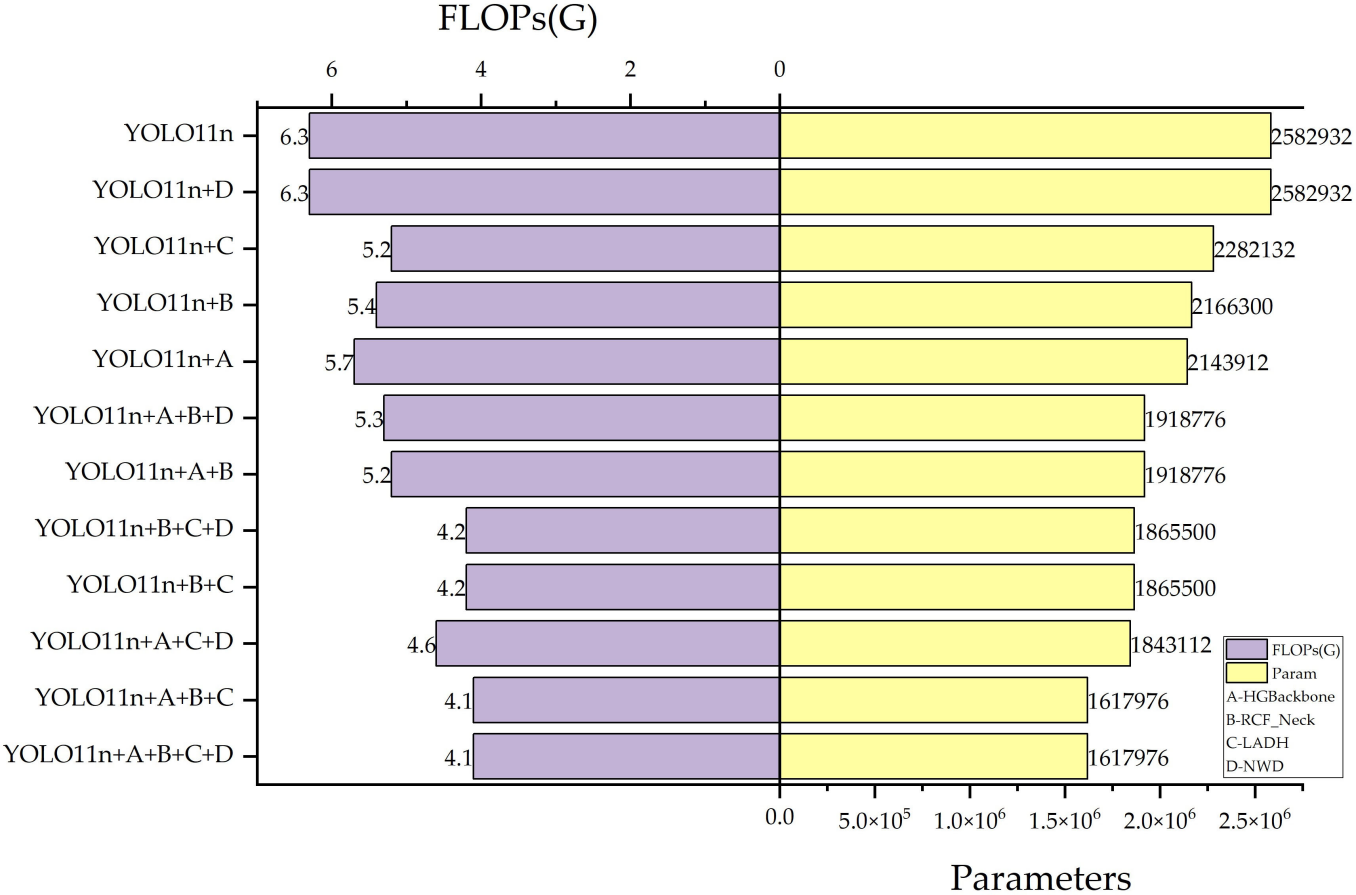
Ablation results comparison.

This study seeks to balance detection precision with a lightweight architecture. Component-wise contributions are assessed via ablation studies under the same data split and training settings. Both precision metrics (mAP@0.5 and mAP@0.5:0.95) and complexity metrics (FLOPs, number of parameters, and inference speed FPS) are recorded, as shown in **Table 3** and **Figure 10**. In **Table 3**, when the backbone of the baseline YOLO11n model is substituted with the lightweight HGBackbone, mAP@0.5 decreases by 0.1% to 93.1%, while mAP@0.5:0.95 increases to 69.7%. At the same time, FLOPs are reduced to 5.7 G with the parameter count reduced to 2,143,912. This modification greatly reduces model parameters and contributes the most among the four improvements, which suggests that the adoption of a lightweight backbone effectively reduces computational and parameter overhead while preserving stable feature representations. To compensate for the loss in detection precision, a new feature fusion network, RCF_Neck, is introduced to strengthen multi-scale semantic interaction and fully represent apple targets of different sizes and maturity stages. When HGBackbone is combined with RCF_Neck as the backbone and neck, mAP@0.5 increases to 94.0% and mAP@0.5:0.95 reaches 69.3%, while FLOPs and the number of parameters is further reduced to 5.2 G and 1,918,776, respectively. This shows that the model can still achieve precision improvement while continuously reducing computational and parameter costs. To further improve classification and regression quality while maintaining lightweight design, LADH-Head is adopted based on HGBackbone and RCF_Neck. As a result, mAP@0.5 and mAP@0.5:0.95 further increase to 94.2% and 70.1%, respectively, while FLOPs are reduced to 4.1 G and the parameter count decreases to 1,617,976, reaching the lowest values. Relative to the baseline, the parameter count is reduced by 37.4%. Finally, considering that slight pixel-level shifts of predicted boxes in small-sized and densely occluded fruit detection tasks may cause a sharp drop in loss values and lead to regression instability and missed detections, the NWD-Loss is incorporated to optimize the training process. The final model achieves mAP@0.5 of 94.9% and mAP@0.5:0.95 of 70.0%, and each exceeds the baseline model by 1.7%. Relative to the variant equipped only with LADH-Head, mAP@0.5 and mAP@0.5:0.95 decrease by 0.9% and 2.8%, respectively, whereas FLOPs and parameter count drop by 21.2% and 29.1%, respectively. **Figure 10** intuitively shows the impact of different components in the ablation study on the parameter count and computational complexity of the model. Here, A, B, C, and D represent HGBackbone, RCF_Neck, LADH, and NWD, respectively. The parameter axis is displayed in scientific notation due to the large magnitude of the values.

**Table 3:** Ablation experiment results.

HGBackbone	RCF_Neck	LADH	NWD	mAP@0.5(%) ↑	mAP@0.5:0.95(%) ↑	FLOPs(G) ↓	Param ↓	FPS↑
				93.2	68.3	6.3	2582932	277.0
✓				93.1	69.7	5.7	2143912	143.5
	✓			93.6	69.1	5.4	2166300	111.3
		✓		95.8	72.8	5.2	2282132	175.7
			✓	95.3	71.4	6.3	2582932	157.8
✓	✓			94.0	69.3	5.2	1918776	130.7
	✓	✓		93.1	69.1	4.2	1865500	116.8
✓	✓	✓		94.2	70.1	4.1	1617976	148.6
✓	✓		✓	94.7	69.9	5.3	1918776	138.1
	✓	✓	✓	94.8	71.3	4.2	1865500	132.2
✓		✓	✓	95.2	70.9	4.6	1843112	192.6
✓	✓	✓	✓	94.9	70.0	4.1	1617976	193.4

↑ indicates that a higher value is better; ↓ indicates that a lower value is better.

Overall, the complexity reduction is markedly larger than the precision decline, suggesting that the final model strikes a favorable trade-off between detection performance and lightweight design while retaining high FPS.

**Figure 11:**
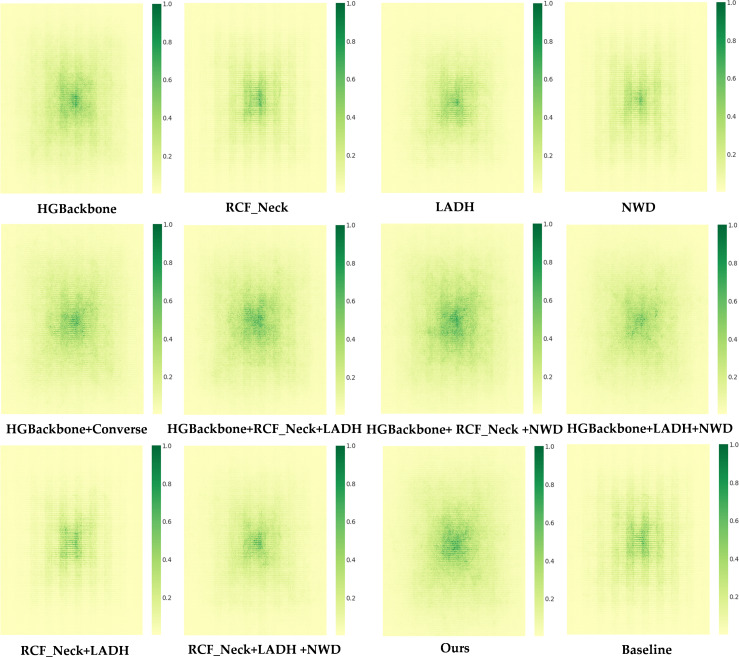
ERP heatmaps under different ablation settings.

Meanwhile, to reveal the influence of each enhanced component on spatial-information aggregation, this study introduces effective receptive field visualization analysis in the ablation experiments. This analysis is used to intuitively show how different structures affect the distribution range of input contributions. The effective receptive field (ERP) heatmaps are plotted as shown in **Figure 11**.

The figure shows the effective receptive field (ERP) heatmaps under different ablation configurations, which are used to describe the contribution of each spatial position in the input image to the output center point. The horizontal and vertical axes correspond to the horizontal and vertical pixel positions of the input image, respectively. The color intensity indicates the normalized contribution magnitude, where darker colors represent a more significant influence on the prediction results. The spatial distribution and diffusion range of high-contribution regions in the heatmaps reflect the size of the effective receptive field as well as the model’s capability for contextual modeling.

From the receptive field visualization results, it can be observed that the high-contribution regions of the baseline results are largely concentrated around the image center and decay rapidly outward, indicating that its effective receptive field is more local. After introducing HGBackbone, the high-contribution region shows a certain outward expansion around the center, indicating that the lightweight backbone enhances mid-scale context aggregation ability. RCF_Neck further strengthens feature propagation, making the high-contribution regions more likely to expand spatially. In contrast, the contribution distribution of LADH is more concentrated, indicating that it mainly improves the discriminative and regression capabilities of the detection head and has a limited effect on receptive field expansion. When RCF_Neck is combined with LADH-Head, the high-contribution regions are the most concentrated, reflecting an emphasis on local effective information under lightweight design. When it is further combined with HGBackbone, the high-contribution regions become more dispersed, indicating a synergistic effect among the backbone, lightweight neck, and detection head. The final model exhibits larger and more continuous high-contribution regions while maintaining lightweight constraints, demonstrating a stronger ability to utilize contextual information.

Based on the analysis of the effective receptive field (ERF) images, this study further conducts a quantitative evaluation using the proportion of effective receptive field area under different contribution thresholds to more objectively compare the effect of each module on context modeling ability. The values reported in the **Table 4** are obtained by averaging the ERF contribution maps of 50 images, and then calculating the minimum area proportion of high-contribution regions required to reach different cumulative contribution thresholds *t*. A smaller *t* indicates that only the most core high-contribution regions are considered, and a larger area proportion means that the model uses a wider range of contextual information earlier during prediction. A larger *t* indicates that nearly all contributions need to be covered, and the corresponding area proportion reflects how dispersed the contributions are and the coverage of long-tail contributions.

**Table 4:** Proportion of effective receptive field area under different contribution thresholds.

Models	*t* = 20%	*t* = 30%	*t* = 50%	*t* = 99%
YOLO11n	4.4%	7.8%	17.4%	95.3%
HGBackbone	4.9%	8.5%	19.0%	95.9%
RCF_Neck	4.2%	7.4%	17.7%	96.5%
LADH	4.2%	7.1%	15.8%	95.9%
NWD	4.7%	8.0%	17.4%	95.9%
HGBackbone + RCF_Neck	5.5%	9.4%	20.3%	95.9%
HGBackbone + RCF_Neck+LADH	5.2%	8.9%	19.2%	95.4%
HGBackbone + RCF_Neck +NWD	5.5%	9.5%	19.8%	95.3%
HGBackbone +LADH+NWD	4.5%	7.8%	17.7%	95.9%
RCF_Neck+LADH	3.4%	6.2%	14.4%	95.9%
RCF_Neck+LADH +NWD	4.5%	7.8%	17.6%	95.9%
Ours	5.3%	9.1%	20.1%	96.0%

Overall, the baseline YOLO11n achieves area proportions of 4.4%, 7.8%, and 17.4% at *t* = 20%, 30%, and 50%, respectively, indicating that it mainly relies on local central regions. After introducing HGBackbone, the values increase to 4.9%, 8.5%, and 19.0%, showing that the lightweight backbone can expand mid-scale context aggregation. Compared with individual modules, combined structures bring more obvious expansion effects. For example, when HGBackbone is combined with RCF_Neck, the area proportion reaches 20.3% at *t* = 50%, increases to 5.5% at *t* = 20%, 9.4% at *t* = 30%, respectively. This indicates that the synergy between the backbone and feature fusion can more effectively propagate useful information to a larger spatial range. In contrast, when the lightweight neck RCF_Neck is combined with the detection head LADH-Head, the area proportions are the lowest, at only 3.4%, 6.2%, and 14.4%, indicating that the lightweight neck makes the contributions more concentrated while reducing computational cost. However, after introducing NWD-Loss, the values at different thresholds increase to 4.5%, 7.8%, and 17.6%, respectively, showing that NWD-Loss can alleviate excessive concentration and enhance mid-scale contribution coverage. More importantly, the final model Ours achieves better effective receptive field coverage under lightweight constraints. At *t* = 50%, it reaches 20.1%, which is close to the best result in the table and significantly higher than the baseline value of 17.4%. This indicates that the model can utilize a wider range of input context when achieving half of the cumulative contribution, which is more beneficial for maturity recognition that depends on overall color distribution and environmental contrast. At the same time, it reaches 96.0% at *t* = 99%, the highest value in the table, indicating more sufficient coverage of long-tail contributions and more stable global information support under complex backgrounds and reflective occlusion conditions. Overall, these quantitative results verify that the proposed model can maintain the advantages of a lightweight structure while still achieving strong context modeling capability and a larger effective receptive field.

### 3.3 Model comparison

To assess the effectiveness and deployment suitability of the proposed method for apple maturity detection, this study selects representative detection frameworks for comparison experiments. These include the classical region proposal–based detector Faster R-CNN (VGG16), several lightweight YOLO series models, and end-to-end detectors from the RT-DETR series. All models are evaluated on the same dataset under a unified evaluation protocol. The detection precision was measured by mAP@0.5, mAP@0.5:0.95 and Precision, Recall, F1-score and the FLOPs (floating-point operations), parameter quantity and inference speed (FPS) were reported to reflect the computational complexity and real-time performance. The comparison results are summarized in **Table 5**.

**Table 5:** Performance comparison of different models.

Models	Precision	F1-score	Recall	mAP@0.5 (%) ↑	mAP@0.5:0.95(%) ↑	FLOPs(G) ↓	Param ↓	FPS ↑
Two stage								
Faster-R CNN(VGG16) (Ren et al., 2017)	70.8	0.76	82.9	75.5	50.0	127.0	119545856	36.2
One stage								
YOLOv8n (Jocher et al., 2023)	85.6	0.85	85.1	90.1	65.4	8.1	3006428	199.0
YOLOv10n (Wang et al., 2024)	88.3	0.86	83.6	90.4	65.4	6.5	2695976	224.7
YOLO11n (Khanam and Hussain, 2024)	90.5	0.88	86.1	93.2	68.3	6.3	2582932	277.0
YOLO12n (Tian et al., 2025)	86.5	0.85	85.2	91.4	66.5	5.8	2509124	240.7
YOLO13n (Lei et al., 2025)	87.1	0.87	86.9	92.3	68.4	6.1	2448675	219.8
Ours	92.8	0.91	89.4	94.9	70.0	4.1	1617976	193.4
End to End								
YOLO26n (Qiu, 2026)	82.2	0.82	83.1	87.4	61.3	5.2	2495864	221.8
DFINE-n (Peng et al., 2024)	84.2	0.81	79.0	87.2	62.8	7.1	3724207	172.8
RT-DETR-r18 (Zhao Y. et al., 2024)	88.3	0.87	86.2	89.7	65.3	57.0	19876896	75.6
RT-DETR-r50 (Zhao Y. et al., 2024)	87.4	0.87	85.4	90.2	55.7	129.6	41962328	51.0
RT-DETR-r101 (Zhao Y. et al., 2024)	92.3	0.88	84.6	90.2	66.7	247.1	74663768	40.6

It can be seen from **Table 5** that Faster R-CNN (VGG16) and the end-to-end RT-DETR series do not show overall advantages in this task, although they have certain advantages in terms of precision, their computational complexity and speed overhead are significantly higher. In contrast, the YOLO series performs better overall. Among them, YOLOv8n and YOLOv10n achieve mAP@0.5 values of 90.1% and 90.4%, respectively, with both reaching 65.4% on mAP@0.5:0.95, and it achieved a Precision of 85.6% and 88.3%, demonstrating good detection capability under relatively low computational cost. The precision of YOLO11n is further improved, with mAP@0.5 reaching 93.2% and mAP@0.5:0.95 reaching 68.3%. It also achieved the highest inference speed among these models at 277.0 FPS, and the Precision reached its peak at 90.5% at this point, reflecting a favorable trade-off between precision and inference speed. YOLO12n and YOLO13n achieve mAP@0.5:0.95 values of 66.5% and 68.4%, respectively. Their overall performance is close to that of YOLO11n. However, in terms of recall rate, YOLO13n has a slightly better ability to reduce missed detections compared to YOLO11n. In addition, YOLO26n and DFINE-n achieve 87.4% and 87.2% mAP@0.5, 61.3% and 62.8% mAP@0.5:0.95, and Precision values of 82.2% and 84.2%, respectively. Although these two models maintain relatively competitive inference speed or moderate complexity, their overall detection performance remains inferior to that of the stronger YOLO11-series baselines and the proposed method.

The proposed method demonstrates the clearest lightweight advantage in this comparison while still achieving the best detection performance. From the analysis in the table, the proposed method uses only 1,617,976 parameters and 4.1 G FLOPs, which are 37.3% and 34.9% lower than those of YOLO11n, respectively. Despite this substantial reduction in model size and computational cost, mAP@0.5 reaches 94.9%, mAP@0.5:0.95 reaches 70.0%, and Precision reaches 92.8%, which are 1.7, 1.7, and 2.3 percentage points higher than those of YOLO11n, respectively. This indicates that the proposed architecture can effectively reduce network weights and computational burden without sacrificing detection accuracy, even under stricter IoU thresholds. In terms of F1-score and recall, the proposed method also maintains clear advantages, achieving an F1-score of 0.91 and a recall of 89.4%. Compared with YOLO11n, the F1-score increases from 0.88 to 0.91, and compared with the highest baseline recall of YOLO13n, the recall is improved by 2.5 percentage points. These results further show that the proposed model maintains a better balance between false positives and false negatives under a more lightweight model design. Although its inference speed is 193.4 FPS on an RTX 4090D-based hardware platform, the proposed method still maintains practical real-time capability for orchard deployment. It should be noted that FPS is not determined solely by FLOPs or parameter count, but is also jointly affected by factors such as model architecture and hardware-dependent implementation efficiency. Therefore, although the FPS of the proposed method is lower than the peak speed of YOLO11n, it still demonstrates clear lightweight advantages by further reducing GFLOPs and parameter count while improving detection precision. From the perspective of practical orchard deployment, 193.4 FPS remains sufficient for real-time applications, indicating that the proposed method prioritizes model compactness and computational reduction while still maintaining practical deployment capability, which is consistent with the lightweight design goal of this study.

**Figure 12:**
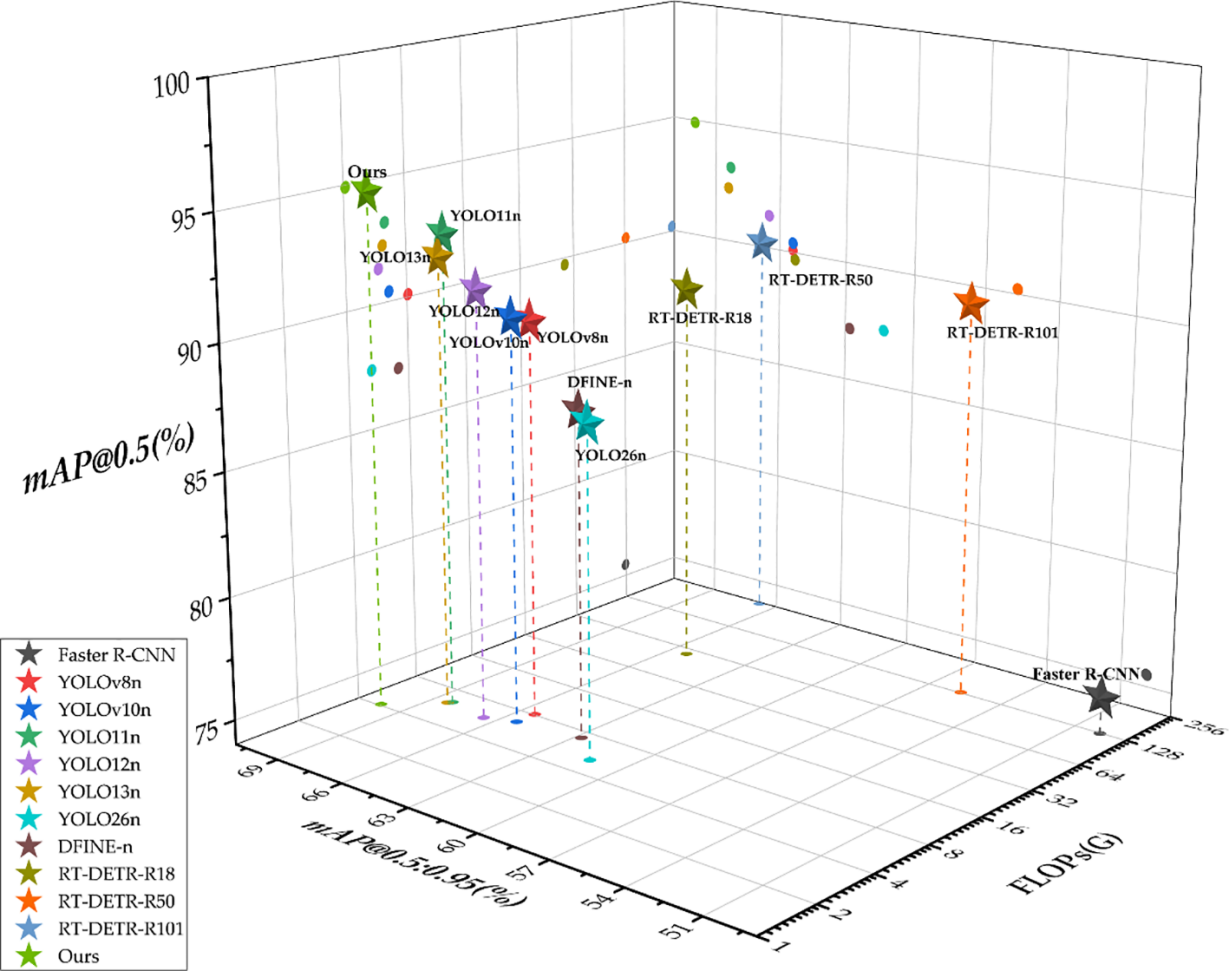
Visualized comparisons across different models.

Additionally, **Figure 12** presents a 3D scatter plot for a more intuitive comparison. It contrasts the proposed method with baseline models using mAP@0.5, mAP@0.5:0.95, and GFLOPs. Pentagrams in different colors represent different models, while dots in different colors indicate the projections of each model onto different planes. Due to the large variation in GFLOPs values, the tick interval on this axis is set in a base-2 logarithmic scale to maintain visual consistency. As shown in the 3D figure, the proposed model located in the upper-left region, which indicates that it achieves both high detection precision and low computational complexity.

In summary, the proposed method achieves superior precision compared with mainstream baseline models, while also providing stronger lightweight characteristics with lower FLOPs and fewer parameters. These findings suggest that the improved architecture better balances precision and computational complexity for apple maturity detection, supporting practical deployment.

Meanwhile, to further demonstrate the lightweight advantage of the proposed model, this study comparisons with state-of-the-art (SOTA) models related to the fruit detection field that focus on lightweight improvements. The comparison is conducted in terms of the number of parameters, GFLOPs, and FPS, as shown in **Table 6**. It can be observed that most existing improvements are developed based on the YOLO series, which makes them easier to deploy in real orchard harvesting scenarios. The results suggest that the proposed model achieves improved lightweight efficiency with fewer parameters and lower floating-point computation. It should also be noted that, from the perspective of deployment-oriented lightweight metrics, the difference between the proposed method and BGWL-YOLO is relatively limited, which is understandable because both methods are essentially lightweight improvements developed on YOLO11n for orchard-related tasks. Therefore, it is reasonable that the two models exhibit a similar parameter scale. However, the optimization paths emphasized by the two methods are not the same. BGWL-YOLO mainly adopts a more efficiency-oriented strategy, such as BiFPN-based feature fusion and GhostConv-based operator replacement, and these designs are usually more likely to translate directly into an advantage in inference speed. In contrast, the proposed HRLN-YOLO places greater emphasis on preserving fine-grained cues related to maturity recognition and enhancing detail-sensitive multi-scale feature fusion through HGBackbone, RCF_Neck, LADH-Head, and NWD-Loss. As a result, while maintaining a parameter scale very close to that of BGWL-YOLO, HRLN-YOLO further reduces GFLOPs from 5.3 G to 4.1 G, reduced by 22%, whereas BGWL-YOLO shows a slight advantage in FPS (197.4). In other words, the two methods reflect different lightweight design priorities: BGWL-YOLO is relatively more speed-oriented, while HRLN-YOLO is more oriented toward balancing lightweight design, fine-grained maturity perception, and robustness under complex orchard conditions. Overall, the proposed method demonstrates competitive overall performance among related lightweight models, and should be understood as a balanced solution for complex-scene perception that integrates lightweight design, detection accuracy, and robustness, rather than as a mere lightweight upgrade.

**Table 6:** Comparison with SOTA models.

Models	Param	FLOPs(G)	FPS	Model Size (MB)
BGWL-YOLO (Qiu et al., 2025)	1620202	5.3	197.4	3.4
YOLO11-ARAF (Lin et al., 2025)	2841453	7.3	76.1	
BMDNet-YOLO (Sun and Wang, 2025)	1800000	5.1	--	--
SR-YOLO (Zhao et al., 2025)	2300000	5.8	--	--
YOLO11-WAS (Du et al., 2025)	1992953	5.0	243.9	--
Ours	1617976	4.1	193.4	3.5

**Figure 13:**
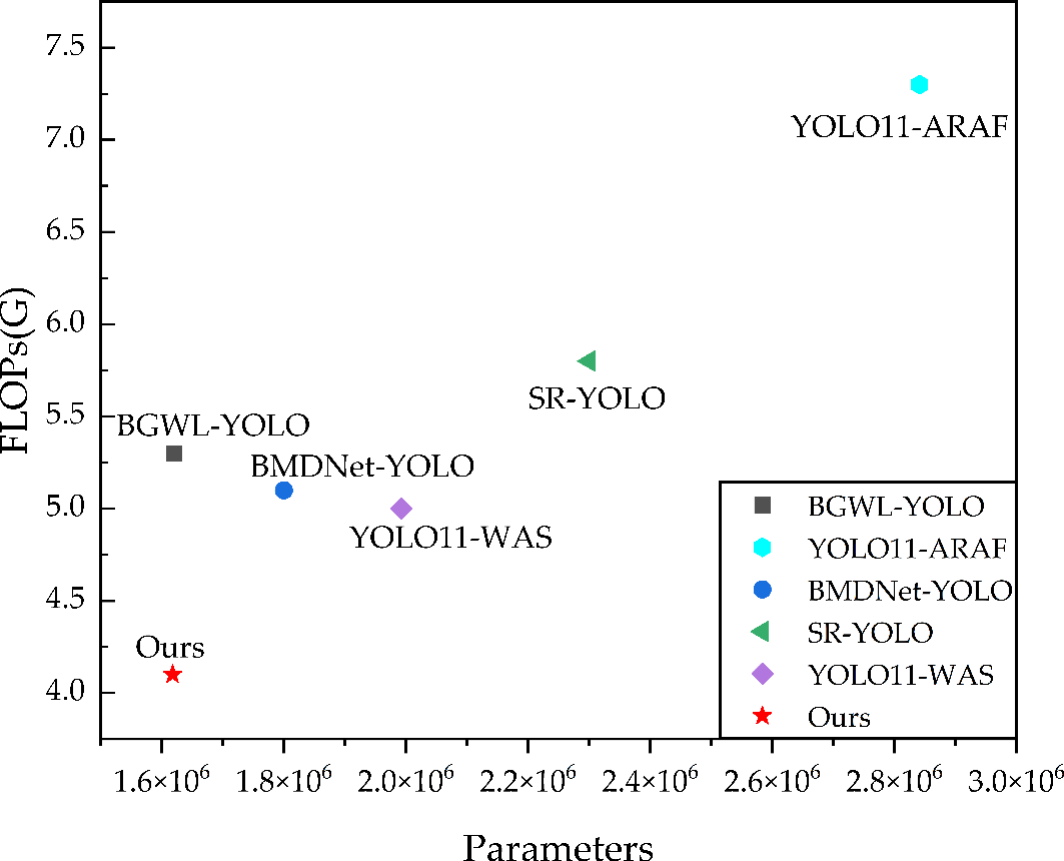
Visualization of the comparison with SOTA models.

**Figure 13** presents a visualization comparing the proposed model with SOTA models. The x-axis is displayed in scientific notation due to the large magnitude of the values.

### 3.4 Model visualization

**Figure 14:**
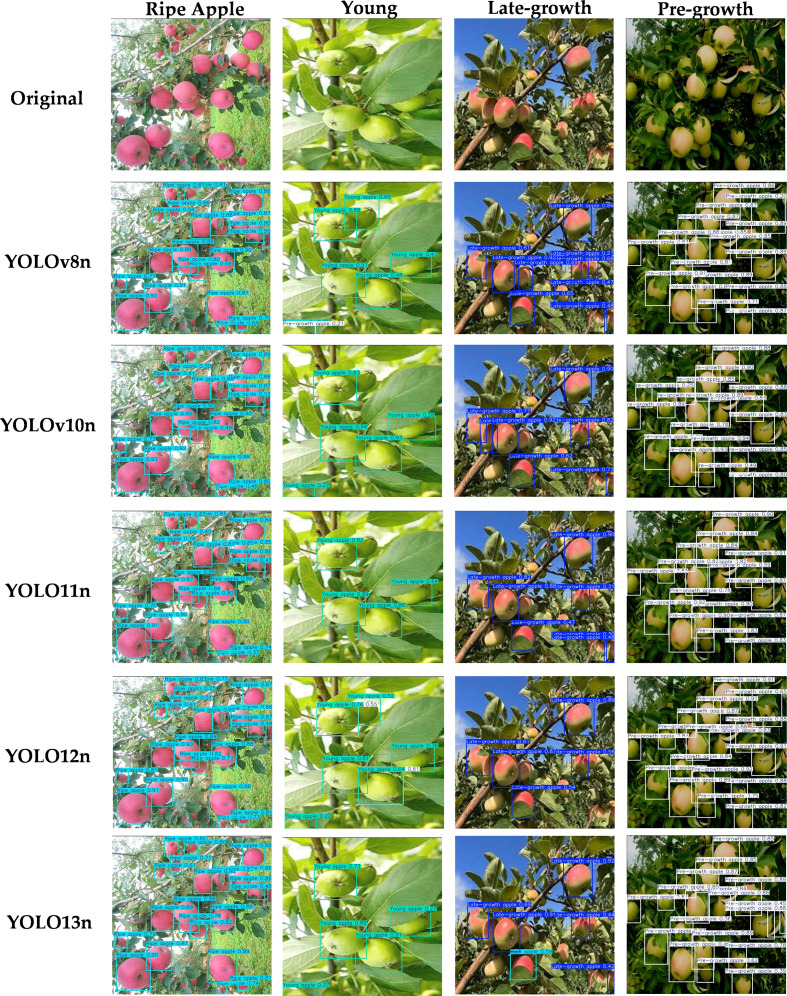
Visualization comparison of different models.

**Figure 15:**
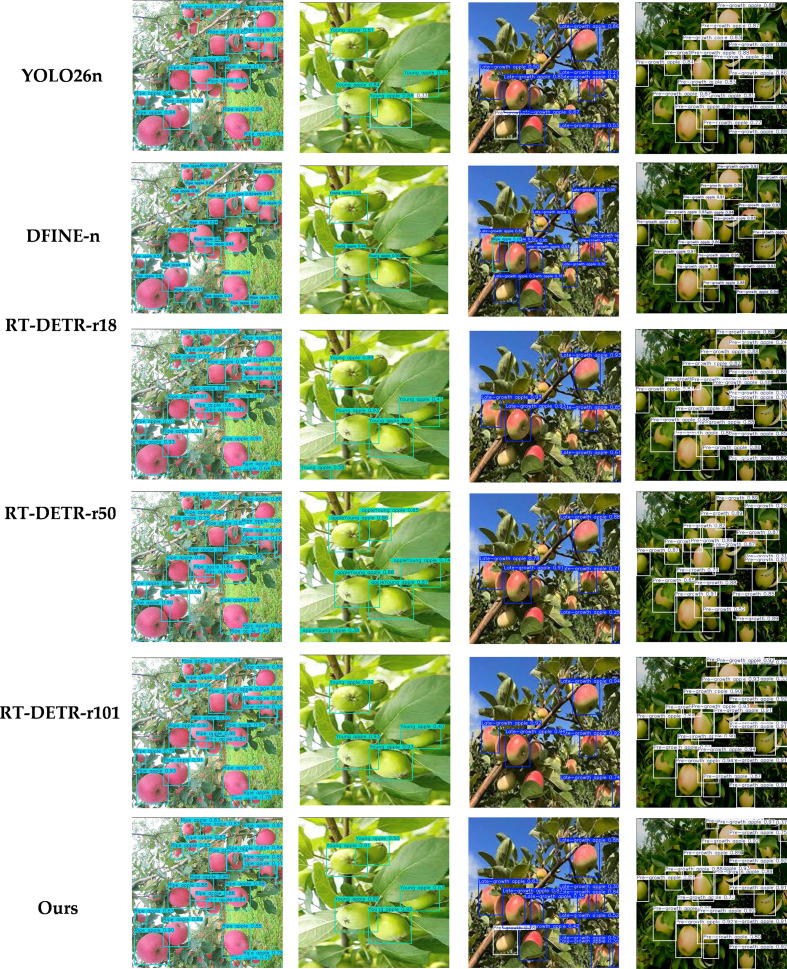
Visual comparison of model detection results under different weather conditions.

As shown in **Figures 14** and **15**, this study presents (column-wise) visualization results of four apple categories at different growth stages (ripe apples, young apples, late-growth apples, and pre-growth apples). In each scene, various external environmental factors are present, including typical interference factors such as occlusion, shadows, small targets, and dense overlaps. Although some samples involve complex conditions such as target occlusion or illumination shadows, the proposed model still maintains a strong response to the targets. In dense scenes of ripe apples and pre-growth apples, the model can still achieve relatively stable detection responses for fruits that are occluded by branches and leaves or only partially visible. In scenes of young apples and late-growth apples, it can be observed that the proposed model effectively enhances its ability to reduce missed detections and false detections on images containing shadow regions caused by illumination variations, as well as on images captured from different viewpoints, demonstrating good scale adaptability and context utilization capability. In contrast, some baseline methods do not perform well under interference conditions. Nevertheless, a few missed detections and false detections can still be observed in highly occluded or densely overlapped scenes. Overall, in most scenes, the proposed method outputs a relatively larger number of detection boxes, showing a certain advantage: while maintaining lightweight deployment efficiency, it achieves stronger detection performance and cross-scene adaptability, and it also shows good stability in controlling false positives and missed detections.

**Figure 16:**
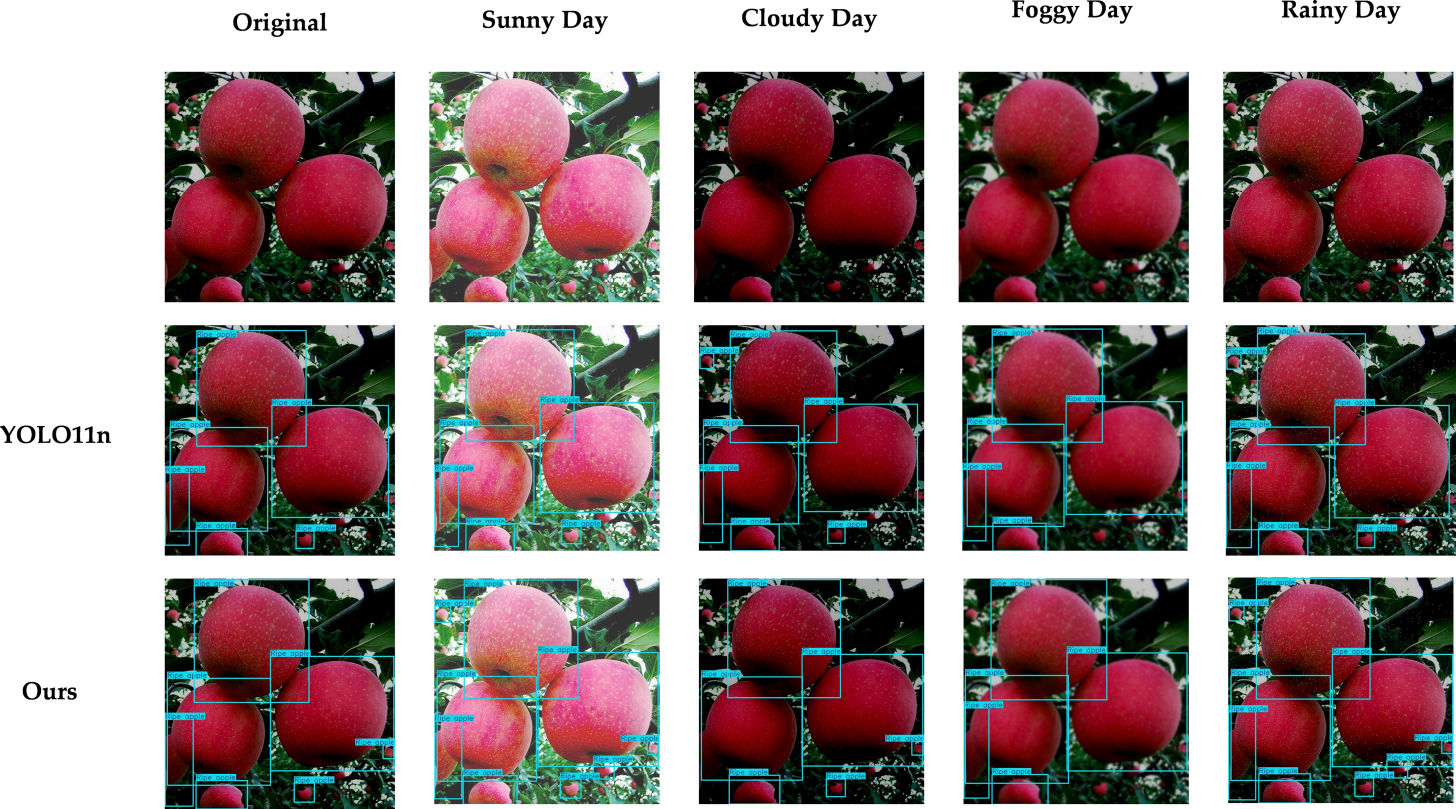
Visual comparison of model detection results under different shooting brightness levels.

As shown in **Figure 16**, to demonstrate the capability of the proposed model to handle different real-world scenarios, this study simulates four typical weather conditions (e.g. sunny, cloudy, foggy, and rainy) based on data augmentation, and compares the results with YOLO11n under the same visualization settings. The proposed model shows relatively stable detection performance under different weather conditions. In low-contrast scenarios such as cloudy and rainy weather, it can still detect visible fruits more sufficiently, with prediction boxes that provide more complete coverage. In foggy weather, imaging blur and reduced contrast can cause some missed detections; however, the overall impact on the proposed model is smaller, and it can still maintain better detection consistency. Under foggy and rainy conditions, a few missed detections may still occur for small or heavily occluded fruits. Through visualization under these four classic weather simulations, the relative trend and stability of the proposed model under different weather disturbances are presented.

**Figure 17:**
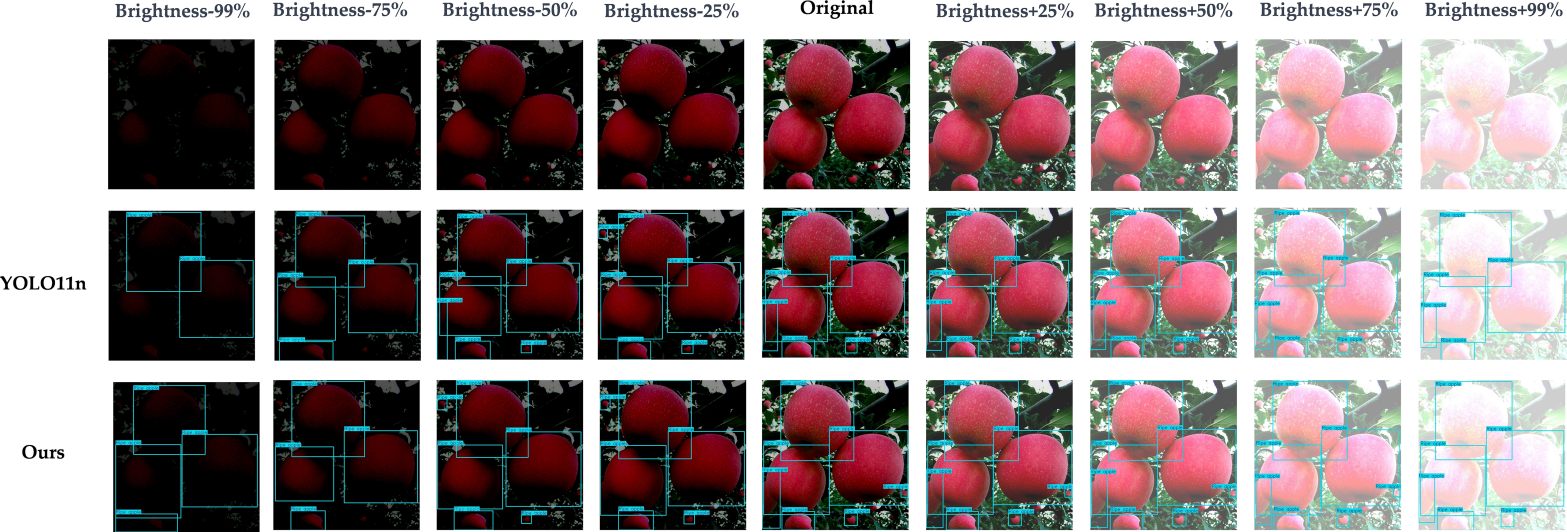
Heatmap comparison of the improved model.

Meanwhile, we considered that when the weather changes or other external factors vary, such as leaf occlusion or shooting angle, the images collected by the harvesting equipment may undergo different brightness changes. Therefore, in order to verify the stability of our model under such conditions, that is, its adaptability to brightness changes in photos, we used online data augmentation to set different brightness-change tests with 25% as one level. The specific results are shown in **Figure 17**. The results show that, as the image becomes darker, image details and texture information are compressed, which leads to an increased possibility of missed detections. When the image is too bright, overexposure will cause interference, and missed detections or unstable predictions can still occasionally be observed. However, compared with YOLO11n, the proposed model performs more stably at all brightness levels, verifying that the model has stronger adaptability to brightness changes in photos, which further verifies the stability of the model.

### 3.5 Heatmap analysis

**Figure 18:**
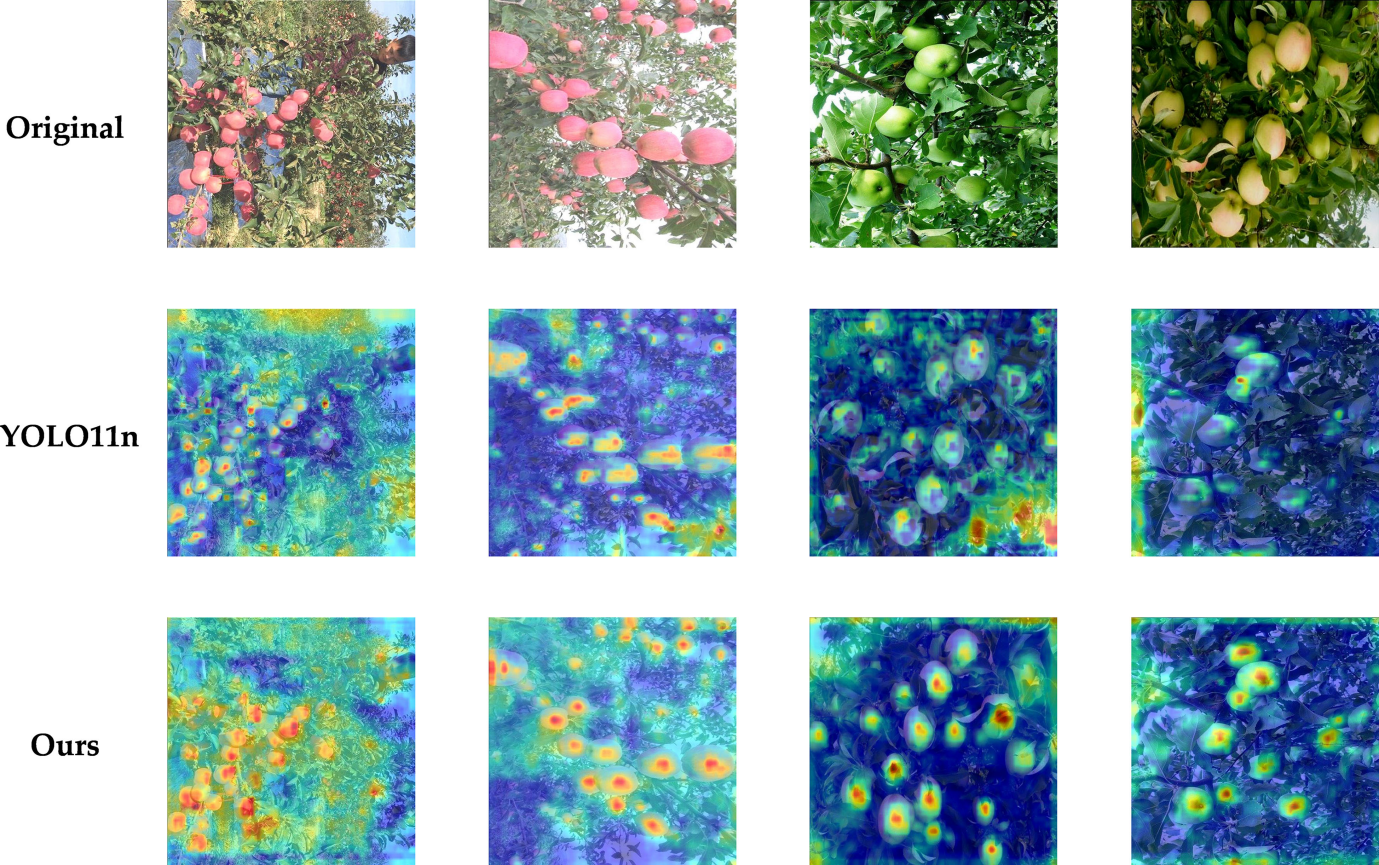
Heatmap comparison of the improved model.

To further examine model attention to apples across different scenes and maturity stages, this study adopts Grad-CAM++ to visualize feature activations. This technique can better illustrate where the model focuses its attention, especially in scenes with target overlap and multiple objects. As shown in **Figure 18**, HRLN-YOLO can allocate attention more accurately to target regions, which can be observed from both the activation intensity and the activation coverage of key areas. Compared with the baseline model YOLO11n, which shows more scattered attention and attention points shifted away from target locations, the proposed HRLN-YOLO demonstrates stronger capability in capturing meaningful features while suppressing background noise. This improves model effectiveness in real orchard settings with cluttered backgrounds or densely distributed targets.

In addition, based on the heatmap analysis, the previously observed missed detections and false detections can be further interpreted. The results reveal that most errors arise in leaf-occluded areas or stem from false detections caused by variations in fruit viewing angles. In such cases, even with the improved detection head, it is still difficult to extract sufficient cues from some fine details, which leads to missed and false detections. More specifically, when the visible region of a fruit is extremely limited, the model attention tends to be partially shifted toward surrounding leaves, branches, or background structures, which weakens the discriminability of maturity-related color and texture information. Similarly, under viewpoint changes or partial exposure, the shape and surface appearance of fruits may become ambiguous, causing confusion between true targets and background-like regions. These observations indicate that the remaining errors are mainly associated with insufficient fine-grained feature perception under severe interference, rather than a failure of the overall detection framework. This also motivates future research toward multimodal approaches and the extraction of richer semantic information to obtain more localization cues and achieve more accurate recognition of target features.

## 4 Discussion

This study proposes a lightweight and efficient detection model, HRLN-YOLO. In the backbone part, we designed a new HGBackbone. It not only improves the modeling ability for texture and edge information, but also reduces unnecessary computation. In this way, detailed features such as apple color and texture can be better passed to the neck. In the feature fusion part, we designed a new RCF_Neck. With the help of the C3K2D module and the reconstruction-enhanced fusion mechanism, it not only keeps the network lightweight, but also improves the fusion ability of fine features such as apple color, texture, and fruit edges. In the detection head, we introduced a highly lightweight detection head, LADH-Head. This head greatly reduces the number of parameters and the computational cost, while also improving detection stability. Finally, we adopted NWD-Loss. By integrating the normalized Wasserstein distance similarity measure into the bounding box regression loss, it improves the regression instability in dense or small-size apple detection. Experimental results show that HRLN-YOLO reduces the number of parameters by 37.3%, lowers the computational complexity by 34.9%, and improves the average precision by 1.7%. Considering other advanced studies on lightweight improvement, the BGWL-YOLO method proposed by (Qiu et al., 2025) greatly reduces model complexity, but its detection accuracy is basically the same as that of the baseline model. The YOLO11-WAS method proposed by (Du et al., 2025) improves accuracy by 2.7% compared with the baseline model, but the reductions in the number of parameters and computational complexity are relatively limited. In contrast, HRLN-YOLO further reduces model parameters and computational complexity by using lightweight design in both the feature extraction module and the feature fusion module, while still improving detection accuracy. Although the inference speed of HRLN-YOLO is lower than that of YOLO11n, it still has practical real-time ability for orchard deployment. For edge-oriented smart orchard applications, model compactness, computational burden, and robustness in complex scenes are often as important as inference speed. From this point of view, this reflects the original purpose of this study, that is, to give priority to lightweight improvement while ensuring accuracy. Therefore, this model shows better overall lightweight performance.

The HRLN-YOLO maturity detection model can be used to detect apples in four different maturity stages, namely Young apple, Pre-growth apple, Late-growth apple, and Ripe apple. In actual orchard work, apple maturity detection can provide managers with more complete maturity information. Based on the information collected by edge devices, managers can make harvesting routes according to the distribution of maturity levels, or pick fruits at specific maturity stages. This not only improves orchard production efficiency, but also optimizes the management mode and promotes the development of smart orchards.

However, the proposed method still has some limitations. First, although the model shows clear advantages in parameter efficiency and computational complexity, its inference speed is not the highest among all compared models. This shows that reducing model complexity and the number of parameters does not always lead to a proportional increase in running speed. Second, according to the qualitative analysis and visualization results, missed detections and false detections still appear in areas with serious leaf occlusion and large viewpoint changes. At the same time, because of device limitations, this study only verified the adaptability of the model to image brightness. More problems that exist in real situations, such as shadows and overexposure caused by different light intensity and incident angles, still need further verification. In highly complex orchard scenes, finer maturity features may still be difficult to capture completely. Future work should further improve the model in two aspects. On the one hand, deployment should be optimized to speed up inference. On the other hand, more data samples from different scenes should be collected, such as images from multiple viewpoints or images with overexposure and shadows, so as to improve the model’s feature perception ability in richer scenes.

## 5 Conclusions

This paper presents a novel lightweight detection framework built upon YOLO11n for identifying apple maturity in orchards. Through the integration of HGBackbone, RCF_Neck, LADH-Head, and the NWD-Loss function, the enhanced HRLN-YOLO attains a 1.7% increase in average precision over YOLO11n, while simultaneously cutting down the number of parameters by 37.3% and computational complexity by 34.9%. When benchmarked against recent lightweight optimized models, the proposed architecture still demonstrates distinct superiority regarding parameter efficiency and computational cost. The lightweight and efficient model proposed in this study can achieve precise detection of apple maturity, effectively optimizing harvest time, reducing resource waste, and lowering post-harvest losses, which has a direct significance for promoting the development of smart agriculture.

## Data Availability

The original contributions presented in the study are included in the article/supplementary material. Further inquiries can be directed to the corresponding author.
